# Estimated Dose-Dependent Associations Between Dietary Intake and Stress Responses in Adolescents and Young Adults: Evidence from a Regional Observational Study in Japan

**DOI:** 10.3390/nu18162612

**Published:** 2026-08-10

**Authors:** Makoto Hazama, Hiroyo Kagami-Katsuyama, Naohito Ito, Mari Maeda-Yamamoto, Hiromi Kimoto, Jun Nishihira

**Affiliations:** 1 Department of Medical Management and Informatics, Hokkaido Information University, Ebetsu 069-8585, Japan; m-hazama@do-johodai.ac.jp (M.H.); k.katsuyama@do-johodai.ac.jp (H.K.-K.); n_ito@do-johodai.ac.jp (N.I.); 2 Institute of Food Research, National Agriculture and Food Research Organization, Tsukuba 305-8642, Japan; yamamoto.mari733@naro.go.jp (M.M.-Y.); kimoto.hiromi604@naro.go.jp (H.K.)

**Keywords:** diet and mental health, dose-dependent relationship, structural equation modeling, adolescents and young adults

## Abstract

**Background/Objectives**: The relationship between diet and mental wellbeing is bidirectional: nutritional psychiatry examines effects of diet on mental health, whereas emotional-eating research focuses on the reverse. Although dose–response links between nutrient intake and health outcomes are well studied, few have analyzed such relationships between food-group intake and stress. Using cross-sectional data from a regional observational study of Japanese adolescents and young adults (males: *n* = 48, age = 32.3 ± 5.23; females: *n* = 124, age = 29.8 ± 5.77), we estimated dose–response associations between intake frequency of various food groups and stress responses. **Methods**: Mental and physical health was assessed using the response values for the mental and physical stress-reaction items in the Brief Job Stress Questionnaire. Intake frequency for each food group was obtained from a food frequency questionnaire (*FFQ*). Although the *FFQ* provides ordinal frequency categories, we demonstrate that structural equation modeling allows these data to be treated as integer values representing monthly intake frequency, enabling the estimation of associations with stress responses. **Results**: The analysis showed that, in females, the presence or absence of intake—rather than the frequency of intake—of dark-green and yellow vegetables was associated with the magnitude of stress responses. In contrast, for bread, noodles, and starchy foods, a monotonic positive association was observed between intake frequency and fatigue. **Conclusions**: Although these associations do not disentangle the bidirectional causal relationship between diet and mental–physical health, they provide evidence on the linear and nonlinear associations between food-group–specific intake frequencies and stress responses in the AYA population.

## 1. Introduction

Over the past several decades, adolescents and young adults (AYA) in high socio-demographic index countries have exhibited a higher prevalence of mental disorders compared with other age cohorts. Furthermore, irrespective of socio-demographic index classification, the proportion of disability-adjusted life years attributable to mental disorders has shown an upward trend among the AYA population [1]. According to data from 2019, an estimated 14.7% of individuals aged 15 to 19 and 14.1% of those aged 20 to 24 worldwide were experiencing mental disorders [2]. When considering self-care as a strategy for stress management among AYA, the relationship between dietary habits and mental health [3] constitutes a particularly salient area of interest.

The relationship between diet and mental health is inherently bidirectional. Influences from the former to the latter operate through pathways such as inflammation and neuroimmune function, the gut–brain axis, neurotransmitter synthesis, epigenetic regulation, and metabolic or glycemic control. Conversely, influences from the latter to the former are mediated by multiple neurobiological and psychological mechanisms, including activation of the hypothalamic–pituitary–adrenal (HPA) axis, autonomic and reward-system processes, neuroendocrine shifts, alterations in interoceptive processing, and psychological mechanisms such as emotional eating [4,5,6,7,8,9,10,11]. Importantly, several of these pathways do not operate in isolation but act as mutual modulators; for example, neuroendocrine shifts can influence autonomic and reward-system activity, while alterations in interoceptive processing can modulate emotional eating and other psychologically mediated responses [5]. Although the causal relationship between diet and mental well-being is bidirectional, the fact that diet represents a powerful modifiable risk factor for human health underscores the value of accumulating evidence—across all levels of evidence—on the quantitative relationship between dietary patterns and mental health conditions [12].

Systematic reviews and meta-analyses examining the quantitative relationship between diet and mental health have predominantly focused on specific nutrients or food constituents [4,7,13,14,15,16,17]. In addition, some studies—partially overlapping with the former group—have investigated particular aspects of diet, such as dietary patterns (e.g., the Mediterranean diet or ketogenic diet), ultra-processed food, or dietary diversity, and their associations with mental health outcomes [5,6,13,17,18,19,20]. The relative abundance of nutrient-based and dietary-pattern–based studies, contrasted with the scarcity of food-group–based research review, may reflect the central role of dietary therapy in clinical medicine, where interventions are often framed in terms of nutrients or structured dietary patterns. However, in community-dwelling or subclinical contexts, improvements in diet should encompass not only nutrients and dietary patterns but also actionable changes at the level of food groups, which represent a more practical and accessible unit for behavioral modification. Against this background, the aim of the present study is to estimate the dose-dependent relationship between food-group consumption and mental health using the limited data resources available.

This study estimates the dose–response relationships between food-group–specific intake frequencies and stress responses using data from a food frequency questionnaire (*FFQ*) and an occupational stress questionnaire collected in an observational survey of adolescents and young adults in a region of Hokkaido, Japan. Although the *FFQ* provides hierarchical categorical data on food intake frequency, the use of a structural equation model (SEM) that allows the error terms across multiple structural equations to be correlated enables us to estimate the underlying continuous intake frequencies and their associations with stress responses. At the same time, this approach allows for the control of confounding factors in the relationship between intake frequency and stress responses. The dose–response curves derived from the estimated SEM revealed nonlinear associations between food-group intake frequencies and stress responses. For some combinations of food groups and stress-response measures, the dose–response curves exhibited a monotonic increasing or decreasing pattern, indicating that higher intake frequency is associated with consistently higher or lower stress responses. In contrast, other combinations showed L-shaped curves, indicating that beyond a certain intake threshold, additional increases in intake frequency no longer correspond to differences in stress responses. These two distinct curve shapes provide useful information for practical health management.

## 2. Materials and Methods

### 2.1. Data

This study draws on data from a cross-sectional survey conducted in Hokkaido and Miyazaki Prefecture between August and October 2024. For the present analysis, we utilize only the Hokkaido sample. The Hokkaido sample comprises 172 participants (48 males and 124 females), whereas the Miyazaki sample includes 48 participants (24 males and 24 females). Because the substantial asymmetry in sample size between the two regions limits our ability to adequately address potential inter-regional heterogeneity, the analyses in this paper are restricted to participants from Hokkaido. In the Hokkaido study, individuals residing in Ebetsu City and its surrounding areas were recruited between June and July 2024. Recruitment was based on self-selection through advertisements, whereby interested individuals responded voluntarily to study announcements. The inclusion criterion was Japanese males and females aged 18 to 39 years as of 1 April 2024. The exclusion criteria consisted of pregnancy, possible pregnancy, or breastfeeding; the use of implanted metallic medical devices (including pacemakers, implantable cardioverter-defibrillators, and artificial femoral heads); and the presence of dermatological conditions such as atopic dermatitis or contact dermatitis.

The observational dataset contains a wide range of information obtained from physical examinations, vital signs, blood tests, urine tests, skin assessments, and self-administered questionnaires. In this paper, we primarily use responses from two of the self-administered questionnaires: the Brief Job Stress Questionnaire [21,22] and the Food Frequency Questionnaire (*FFQ*) [23]. We employed the former to obtain measures of mental and physical health, and the latter to derive measures related to dietary habits for our analysis. The questionnaire asked about the respondent’s mental and physical stress responses at the time of answering, while the food intake frequencies referred to the dietary habits over the preceding year.

**Figure A1:**
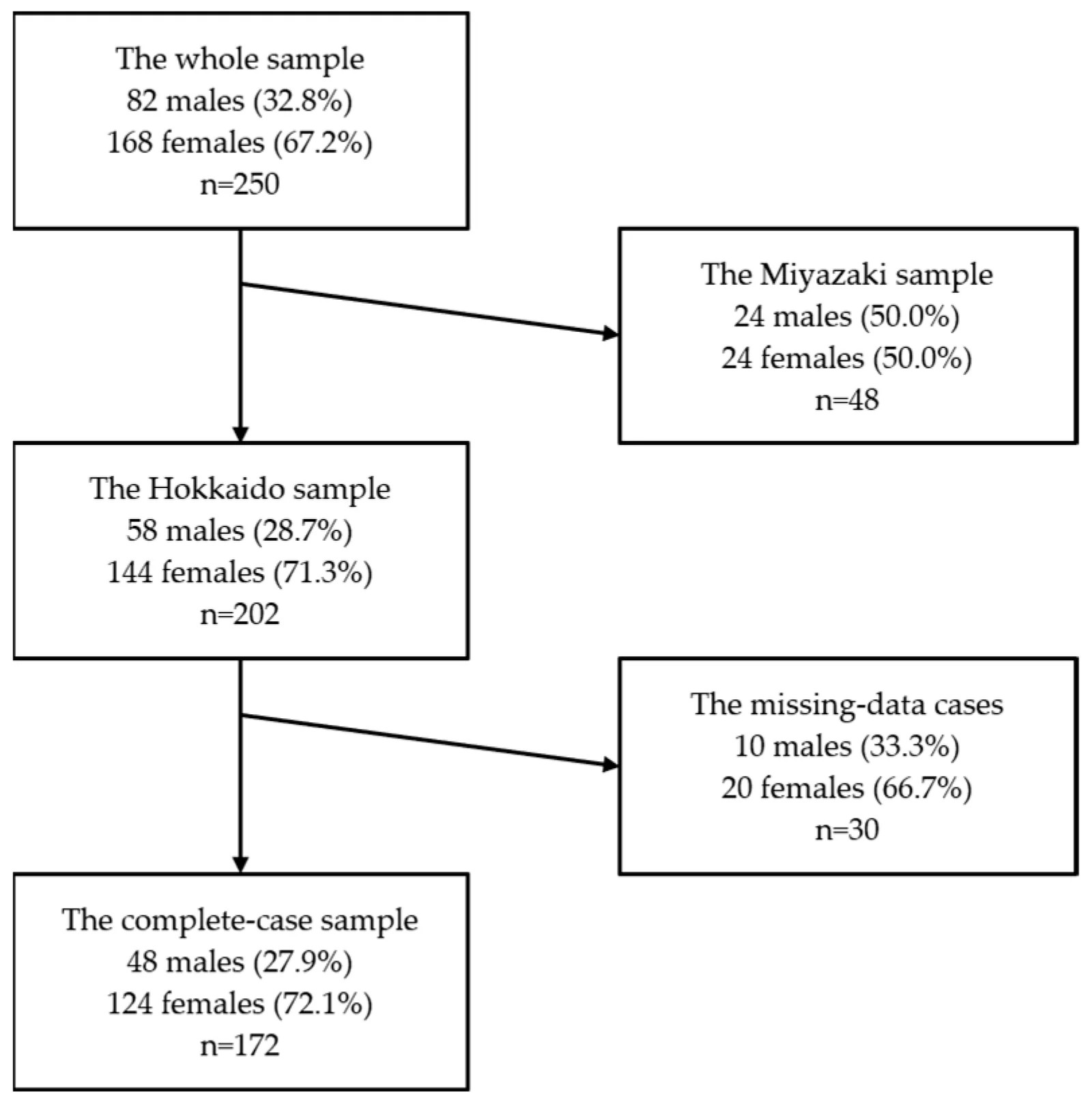
Flowchart of the sample selection.

In addition, we use background information such as gender, age, educational attainment, household characteristics, and employment status, as well as questionnaire responses related to exercise habits. The procedure for extracting the analytical subsample from the overall sample of the observational study is presented in Figure A1 in Appendix A. The results of the comparison between the complete-case and missing-data case samples are presented in Table S1 of the Supplementary Materials. For all variables observed in both samples—age, gender, cohabitation status, educational attainment, and employment status—there are no significant differences between the two samples.

This study was approved by the Ethics Committee of the Hokkaido Information University (approval date: 26 March 2024; approval number: 2023-14), and written consent was obtained from the participants. The research was conducted in accordance with the Helsinki Declaration.

### 2.2. Data Analysis

In this paper, we aim to estimate the relationship between food intake frequency and health outcome variables—that is, the dose–response relationship. However, only stratified data on food intake frequency are available. What this study seeks to clarify is not the relationship based on stratified doses, but rather the relationship based on an unstratified (continuous) dose. To address this limitation, we employ a structural equation model (SEM).

**Figure 1:**
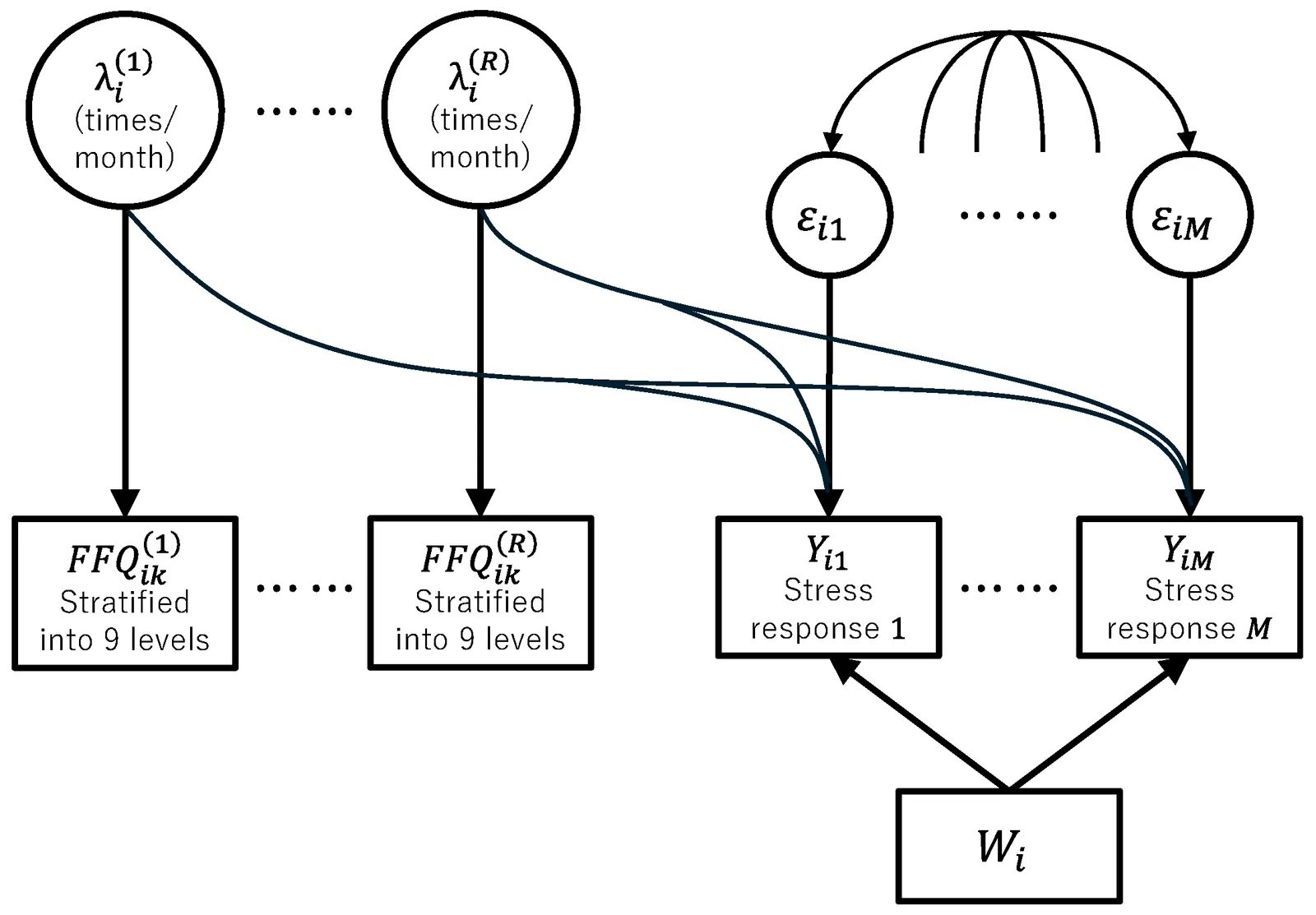
Path diagram of the structural equations model.

Figure 1 presents the path diagram of the model. In the diagram, squares denote observed variables, whereas circles denote unobserved variables. The variable ${FFQ}_{ik}^{\left(r\right)}$ denotes the *FFQ* response for individual i $\left(i=1,\;2,\;\cdots ,\;N\right)$ regarding item k $\left(k=1,\;2,\;\cdots ,\;K_{r}\right)$ within food group r $\left(r=1,\;2,\;\cdots ,\;R\right)$. The *FFQ* responses are stratified into nine categories: 1 (less than once per month), 2 (1–3 times per month), 3 (1–2 times per week), 4 (3–4 times per week), 5 (5–6 times per week), 6 (once per day), 7 (2–3 times per day), 8 (4–6 times per day), and 9 (7 or more times per day). Naturally, there exist underlying unstratified intake frequencies behind these stratified *FFQ* data, but they are unobserved. In the model, these underlying food-specific intake frequencies are assumed to follow a Poisson distribution. The parameter of this Poisson distribution depends on $\lambda_{i}^{\left(r\right)}$, which represents the individual-specific component of the mean intake frequency for food group r. Meanwhile, individuals’ health status is measured by M variables, with $Y_{im}$ denoting the m-th health measure for individual i $\left(i=1,\;2,\;\cdots ,\;N;m=1,\;2,\;\cdots ,\;M\right)$. The health outcomes $Y_{i1},\;\cdots ,\;Y_{iM}$ are decomposed into a part predicted by the covariates $W_{i}$ and the food-group-specific mean intake frequencies $\lambda_{i}=\left(\lambda_{i}^{\left(1\right)},\;\cdots ,\;\lambda_{i}^{\left(R\right)}\right)$, and a residual component $\varepsilon_{i}=\left(\varepsilon_{i1},\;\cdots ,\;\varepsilon_{iM}\right)$. The residual terms $\varepsilon_{i1},\;\cdots ,\;\varepsilon_{iM}$ are allowed to be correlated with one another. Through this model, it is not the direct relationship between the observed *FFQ* responses and the health outcomes Y that is estimated; rather, the relationship between the unobserved λ and the observed Y is estimated, and this relationship is interpreted as the dose–response relationship between food-group-specific intake frequency and health measures.

Broadly speaking, the structural equation model presented in this paper consists of two components that are related in a unidirectional manner: the measurement part and the structural part. The former corresponds to the left half of Figure 1 $\left(\lambda \to FFQ\right)$, while the latter corresponds to the right half $\left(\lambda ,\;W,\;\varepsilon \to Y\right)$. The measurement part represents the relationship between the stratified *FFQ* response values and the pre-stratification food-specific intake frequencies, as described in Equations (1)–(3):

(1) $$\begin{array}{cc} \begin{array}{ccc} {FFQ}_{ik}^{\left(r\right)}=h & \Leftrightarrow & \tau_{h-1}<{\tilde{X}}_{ik}^{\left(r\right)}\leq \tau_{h} \end{array} & \left(h=1,\;2,\;\cdots ,\;9\right) \end{array},$$

(2) $${\tilde{X}}_{ik}^{\left(r\right)}\sim \mathrm{Poisson}\left(\lambda_{ik}^{\left(r\right)}\right),$$

(3) $$\lambda_{ik}^{\left(r\right)}=\lambda_{i}^{\left(r\right)}+\lambda_{k}^{\left(r\right)},$$

where the subscripts i, k, and r denote the individual, the food item, and the food group, respectively. The food groups were classified into ten categories according to a widely used dietary classification framework (Table A1, Appendix B). The food groups with a small number of items are dairy products and eggs (K = 5), soybean products (K = 6), and processed meat and fish products (K = 6), whereas the others range from K = 11 to 24. To ensure analytical feasibility, several small categories were merged with similar groups. As noted above, the *FFQ* response categories consist of nine levels, ranging from “less than once per month” to “seven or more times per day”. Behind these stratified *FFQ* response categories lies the unstratified intake frequency ${\tilde{X}}_{ik}^{\left(r\right)}$, measured as the number of times a given food is consumed per month. The variable ${\tilde{X}}_{ik}^{\left(r\right)}$ is not observed. Because the relationship between the *FFQ* frequency categories and the monthly intake frequency is straightforward, the thresholds τ in Equation (1) can be determined directly from the *FFQ* categories ($\tau_{0}=-\infty ,\;\tau_{1}=1,\;\tau_{2}=3,\;\tau_{3}=11,\;\tau_{4}=20,\;\tau_{5}=28,\;\tau_{6}=46,\;\tau_{7}=107,\;\tau_{8}=198,\;\tau_{9}=+\infty$ times/month). In this model, as shown in Equation (2), the intake frequency ${\tilde{X}}_{ik}^{\left(r\right)}$ of food item k in food group r for individual i follows a Poisson distribution with parameter $\lambda_{ik}^{\left(r\right)}$. As expressed in Equation (3), the parameter $\lambda_{ik}^{\left(r\right)}$ is decomposed into two components: $\lambda_{i}^{\left(r\right)}$, attributable to individual i, and $\lambda_{k}^{\left(r\right)}$, attributable to food item k. Foods that are more frequently consumed by many people in modern Japanese society, such as eggs and milk, tend to have larger values of $\lambda_{k}^{\left(r\right)}$. The component of the mean intake frequency attributable to the individual, $\lambda_{i}^{\left(r\right)}$, is modeled as influencing that individual’s health status; this constitutes the remaining structural part of the model, represented by Equations (4)–(7):

(4) $$\mathrm{Pr}\left(Y_{im}=j|\lambda_{i},\;D_{i},\;W_{i}\right)=\mathrm{Pr}\left(\theta_{m,\;j-1}<{\tilde{Y}}_{im}\leq \theta_{m,\;j}\right),$$

(5) $${\tilde{Y}}_{im}=+{\left(\beta_{0m}+D_{i}\beta_{1m}\right)}^{\prime }\ln\lambda_{i}+\gamma_{m}^{\prime }W_{i}+\varepsilon_{im},$$

(6) $$\mathrm{Corr}\left(\varepsilon_{im},\;\varepsilon_{il}\right)=\rho_{ml},$$

(7) $$\varepsilon_{im}\sim \mathrm{logistic}\left(0,\;1\right),$$

where $\lambda_{i}\equiv \left(\lambda_{i}^{\left(1\right)},\;\cdots ,\;\lambda_{i}^{\left(R\right)}\right)$. As noted above, the variable $Y_{im}$ represents health indicator m for individual i. In the present analysis, we use as health indicators the items related to stress responses in the Brief Job Stress Questionnaire (BJSQ) [21,22]. The stress responses consist of six subscales and a total of 29 items: vigor (three items), irritability (three items), fatigue (three items), anxiety (three items), depression (six items), and physical complaints (11 items). Each item is measured on a four-point Likert-type scale. In the analysis, we calculated scores for each of the six subscales—vigor, irritability, fatigue, anxiety, depression, and physical complaints—based on the items corresponding to each subscale. Following the evaluation method described by [21], these scores were then converted into the following five stress-response levels: 0 = low, 1 = somewhat low, 2 = average, 3 = somewhat high, and 4 = high. The SEM specified in Equations (1)–(7) was estimated using these six subscale scores as outcome variables.

In Equations (4)–(7), the ordinal variable $Y_{im}$, representing the stress response of individual i, is used as the dependent variable in an ordered logit regression model. The latent variable ${\tilde{Y}}_{im}$ is a continuous variable that underlies and determines the ordinal variable $Y_{im}$. In Equations (4) and (5), $W_{i}$ denotes the covariates for individual i, as described earlier; however, gender is treated separately from the other covariates and is represented by the variable $D_{i}$. The variable $D_{i}$ is a dummy variable that takes the value 1 when individual i is female. As shown in Equation (5), the model allows the relationship between the food specific intake frequency $\lambda_{i}$ and the latent stress response variable ${\tilde{Y}}_{im}$ to differ by gender. In addition to gender, the covariate vector $W_{i}$ includes age, body mass index (BMI), a dummy variable indicating whether the individual lives with others, a dummy variable indicating employment status, and dummy variables representing physical activity level [24,25,26].

The primary focus of this study regarding the SEM in Equations (1)–(7) is the dose–response relationship between the intake frequency of each food category $\lambda_{i}^{\left(r\right)}$, and the stress response $\mathrm{Pr}\left(Y_{im}\geq j|\lambda_{i},\;D_{i},\;W_{i}\right)$. As analytical results, we will present dose–response curves for each combination of stress response item m and food category r.

In the analytical framework of this study, the estimation results allow us to draw an interesting distinction regarding the shape of the estimated dose–response curve between the intake frequency of food group r and stress response m. In addition to distinguishing whether the curve slopes upward or downward, we can also distinguish whether the curve exhibits a bend. In other words, the relationship between the intake frequency of food group r and stress response m may differ substantially depending on the level of intake frequency, or it may not. Because the degree of bending in the curve is a continuous quantity, intermediate levels of bending are also possible. We define the measure Bend for the degree of curvature as follows.

(8) $${Bend}_{mr}\equiv \frac{1}{G}\sum_{g=1}^{G}\frac{\mathrm{Pr}\left(Y_{m}|\lambda^{\left(r\right)}=\lambda_{g}\right)-\mathrm{Pr}\left(Y_{m}|\lambda^{\left(r\right)}=\lambda_{1}\right)}{\mathrm{Pr}\left(Y_{m}|\lambda^{\left(r\right)}=\lambda_{G}\right)-\mathrm{Pr}\left(Y_{m}|\lambda^{\left(r\right)}=\lambda_{1}\right)},$$

where $\lambda_{g}\;\left(g=1,\;\cdots ,\;G\right)$ denotes the grid specified for computing the posterior predictive curves of intake frequency.

Equation (8) represents the degree to which the curve drawn by $P\left(Y|\lambda \right)$, as a function of intake frequency λ, deviates from a straight line. Bend has properties similar to those of the Gini coefficient. However, unlike the Gini coefficient, the range of Bend is between 0.5 and 1. When $P\left(Y|\lambda \right)$ is a straight line, Bend takes the value 0.5. When $P\left(Y|\lambda \right)$ deviates maximally from a straight line—that is, when $P\left(Y|\lambda \right)$ increases or decreases with an infinitesimal initial increase in λ, and then remains unchanged despite further increases in λ—Bend takes the value 1. In our analysis, for each combination of food group r and stress response m, we compute the posterior predictive mean and the 90% HDI of Bend and use it as a measure for characterizing the shape of the dose–response curve. We also compute the proportion of posterior predictive samples in which the dose–response relationship is positive and negative, respectively.

Using the data on FFQ, Y, W, and D, we sampled the posterior distributions of the parameters λ, θ, α, β, and γ via Bayesian estimation. Summary statistics for FFQ, Y, W, and D are presented in Tables S1–S3 of the Supplementary Materials. A standard normal distribution was used as the prior for each parameter. Since λ is restricted to positive values, we estimated lnλ instead. Note that if we only had Equation (3), $\lambda_{i}^{\left(r\right)}$ and $\lambda_{k}^{\left(r\right)}$ would not be identifiable, but when combined with Equation (5), the two become identifiable. Although the intake frequency by food category is decomposed into individual factors and food-category factors, the individual factors are extracted as between-person variation associated with stress-response factors. By setting $\theta_{m,0}=-\infty$ and $\theta_{m,J}=+\infty$ and omitting an intercept term on the right-hand side of Equation (5), the threshold parameters θ become identifiable. The burn-in period and the Markov chain Monte Carlo (MCMC) sample size were set to 2000 and 1000 respectively. 4 chains were used to compute the convergence diagnostics.

### 2.3. Computational Tools and Packages

For the analyses, Python (version 3.12.12) was used. In particular, the estimation of the SEM was conducted with the PyMC library (version 5.20.1).

## 3. Results

Descriptive statistics and cross-tabulations for the analytical dataset are presented in Table A2 of Appendix C and Tables S2 and S3 of the Supplementary Materials. Among the covariates—age, BMI, college graduate dummy, cohabitation dummy, employment dummy, and physical activity—the mean values of age, BMI, and the college graduate dummy differ significantly between males and females. Regarding the stress response scale, two of the six items, irritability and fatigue, exhibited significant gender differences. With respect to the *FFQ*, items such as chicken fried and chocolate were consumed more frequently by females, whereas items such as mustard and wasabi were consumed more frequently by males.

The estimation results of the model in Equations (1)–(7) obtained via MCMC sampling, as well as the posterior diagnostics regarding convergence, are presented in Table S4 of the Supplementary Materials. All R-hat (Gelman–Rubin statistic) values based on the four chains are equal to 1, and the effective sample sizes (ESS) are sufficiently large, providing no indication of convergence problems.

**Figure 2:**
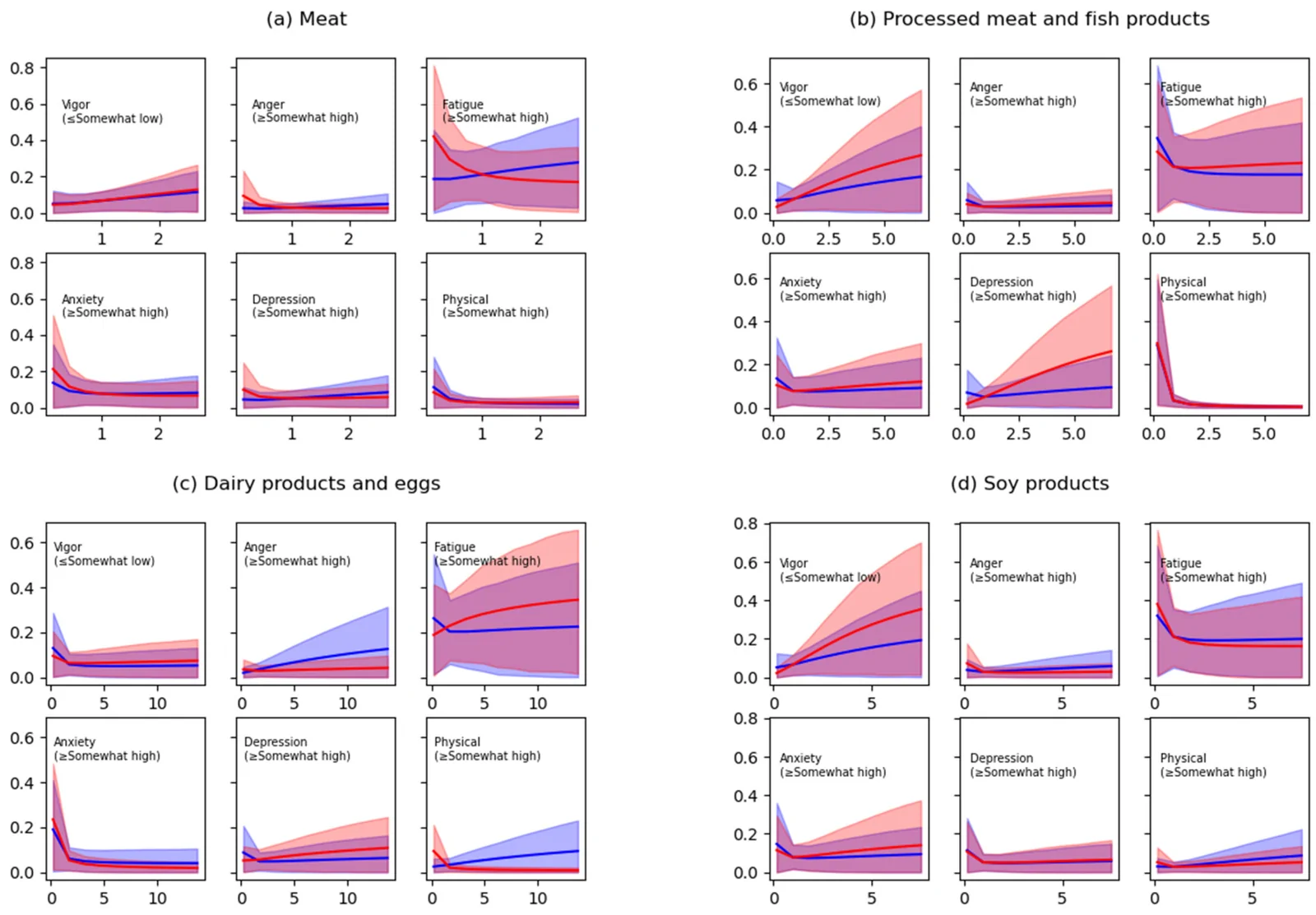

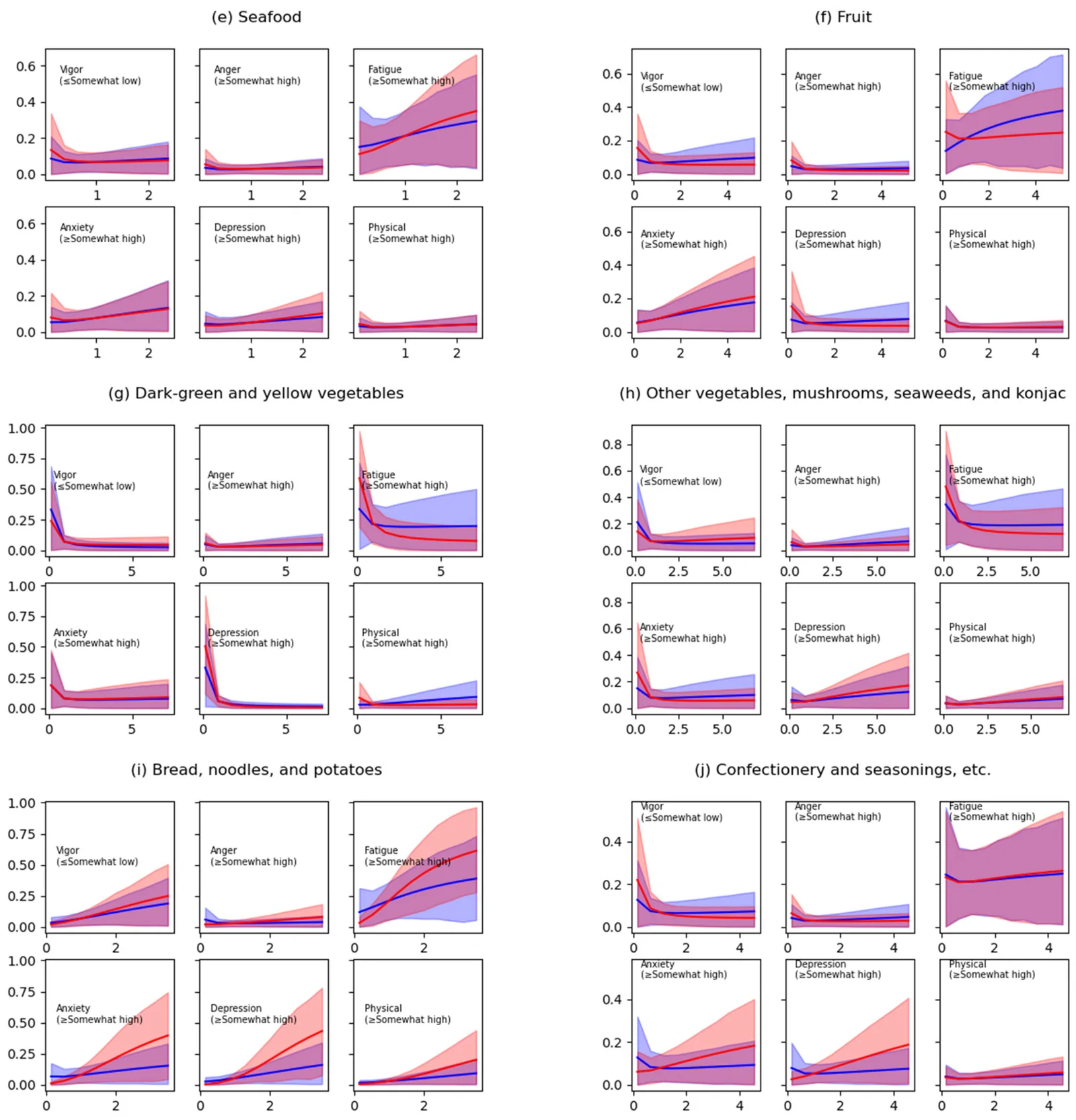
Dose-response curves for each pair of food category and stress response. (**a**) Meat; (**b**) Processed meat and fish products; (**c**) Dairy products and eggs; (**d**) Soy products; (**e**) Seafood; (**f**) Fruit; (**g**) Dark-green and yellow vegetables; (**h**) Other vegetables, mushrooms, seaweeds, and konjac; (**i**) Bread, noodles, and potatoes; (**j**) Confectionary, seasonings, etc. The horizontal axis represents intake frequency (times per month), and the vertical axis represents the probability of being classified as having a strong stress response (with vigor being the only one interpreted in the opposite direction). Red indicates females and blue indicates males. The solid lines show the mean of the posterior predictive distribution, and the bands represent the 90% HDI (highest density interval).

Figure 2 presents dose–response curves for each food category and each type of stress response, based on the posterior predictive means and 90% highest density intervals (HDIs) estimated from the model. In each panel, the horizontal axis represents intake frequency measured as the number of times per month, and the vertical axis represents the probability that the stress scale is judged as “somewhat high” or “high” (for vigor only, “somewhat low” or “low”). The range of the horizontal axis differs across food categories because it reflects the variation in intake frequencies observed in the sample for each category. The color-coded series indicate females in red and males in blue.

Because the sample size is approximately 170, the HDI bands are wide. Consequently, clear differences between males (blue) and females (red) cannot be confirmed. Even so, for each dose–response curve, it is still possible to distinguish whether the trend is increasing, decreasing, or flat, and—if not flat—whether it is linear or L-shaped. The increases or decreases, and overall shapes of the graphs can be identified not only through visual inspection but also by the proportion of posterior predictive curves that exhibit increasing or decreasing patterns, as well as by the curvature measure defined in Equation (8). The values of these measures are presented in Table A3 of Appendix D. For example, among females, a pronounced linear dose–response relationship is observed for the intake of bread, noodles, and potatoes. The proportion of posterior predictive relationships in which the probability of experiencing “Somewhat high” or “High” fatigue increases with intake frequency is 0.98, and an increase of one intake per month is associated with an approximately 17-percentage-point higher probability of greater fatigue (90% HDI = [6 percentage points, 29 percentage points]). Similarly, the proportion of posterior predictive relationships in which the probability of experiencing “Somewhat high” or “High” anxiety increases with intake frequency is 0.98, with an increase of one intake per month corresponding to an approximately 12-percentage-point higher probability of greater anxiety (90% HDI = [0 percentage points, 22 percentage points]). For depression, the proportion of posterior predictive relationships indicating that the probability of experiencing “Somewhat high” or “High” symptoms increases with intake frequency is 1.00, and an increase of one intake per month is associated with an approximately 13-percentage-point higher probability of greater depressive symptoms (90% HDI = [2 percentage points, 24 percentage points]).

In contrast, for females, again, fatigue shows an L-shaped decreasing dose–response curve for dark-green and yellow vegetables (Bend = 0.81, 90% HDI = [0.73, 0.88]), while depression show L-shaped decreasing curves for dark-green and yellow vegetables (Bend = 0.87, 90% HDI = [0.84, 0.90]). The point at which the curve bends is around one time per month. That is, compared with groups that consume almost none of these foods (less than once per month), groups that consume them at least once per month have a lower probability of weaker vigor and a lower probability of stronger fatigue or depression. However, among groups consuming these foods at least once per month, intake frequency is largely unrelated to the probability of each stress-scale judgment.

For females, the results based on the posterior predictive means indicate that consuming dark-green and yellow vegetables is associated with about a 50 percentage points (90% HDI-[10 percentage points, 92 percentage points]) lower probability of reduced depression and about a 50 percentage points (90% HDI-[5 percentage points, 99 percentage points]) lower probability of stronger fatigue.

For males, the smaller sample size relative to females likely contributed, at least in part, to the absence of quantitatively pronounced results, except for the L-shaped relationship observed between the intake of processed meat and fish products and physical stress responses (Bend = 0.87, 90% HDI = [0.82, 0.90]).

## 4. Discussion

This study employed a SEM with error term correlations to estimate dose–response relationships between food-group-specific intake and health outcomes when dietary data are obtained from an *FFQ*. In this framework, individual-level intake frequencies for each food group are estimated by leveraging variation in two directions: variation across individuals and variation across food items. When considering only the measurement part of the model, represented by Equations (1)–(3), the component attributable to individuals, $\lambda_{i}^{\left(r\right)}$, and the component attributable to food groups, $\lambda_{k}^{\left(r\right)}$, are not separately identifiable. However, when combined with the structural part of the model, represented by Equations (4)–(7), the portion of variation that explains differences in stress responses is attributed to $\lambda_{i}^{\left(r\right)}$, thereby enabling the identification of $\lambda_{i}^{\left(r\right)}$ and $\lambda_{k}^{\left(r\right)}$ in the full model. In other words, the present analysis examines the dose–response relationship between health outcomes and the individual-level variation in intake frequency—variation that is distinct from the portion attributable to the food groups themselves (i.e., the spectrum ranging from commonly consumed to rarely consumed foods).

At first glance, the identification strategy described above may appear problematic. If the maximum possible portion of the cross-sectional variation in food-intake frequency were allocated to the prediction of stress responses, it might seem as though the true relationship between food-intake frequency and stress responses would be artificially inflated. However, in reality, no such inflation occurs. This is because the under-identification of $\lambda_{i}^{\left(r\right)}$ and $\lambda_{k}^{\left(r\right)}$ in Equations (1)–(3) arises from additivity: adding an arbitrary constant to one of $\lambda_{i}^{\left(r\right)}$ or $\lambda_{k}^{\left(r\right)}$ and subtracting the same constant from the other yields results that are observationally indistinguishable from the original specification. Replacing $\ln\lambda_{i}^{\left(r\right)}$ with $\ln\left(\lambda_{i}^{\left(r\right)}+constant\right)$ on the right-hand side of regression Equation (5) does not generally lead to an overestimation of the parameter β. In our analysis, $\lambda_{i}^{\left(r\right)}$ is log-transformed because its distribution is long-tailed; however, if the log transformation were not applied, adding an arbitrary constant to $\lambda_{i}^{\left(r\right)}$ in regression Equation (5) would have no effect on the estimation results. In other words, the analytical results are influenced not by the identification strategy itself but solely by the functional specification (i.e., log transformation) of the regression model.

For AYAs in the area surrounding Ebetsu City, Hokkaido, bread, noodles, and potatoes, showed a monotonically increasing dose-dependent association with a risk of fatigue, anxiety, and depression for females. Dark-green and yellow vegetables showed an L-shaped, decreasing dose-dependent association with a risk of fatigue and depression for females, and processed meat and fish products showed an L-shaped decreasing dose-dependent association with a risk of physical stress responses for males. Although these associations were conditioned on confounding factors—including sex, age, BMI, and psychosocial factors—reverse causality cannot be ruled out. Considering the documented effects of foods with strong antioxidant and anti-inflammatory properties (e.g., dark-green and yellow vegetables, seaweeds, mushrooms) on mental well-being [27,28,29,30], as well as the tendency for the intake of high-sugar and high-fat foods to increase under stress eating [20,31], the above findings may be interpreted as reflecting the stress-mitigating effects of dark-green and yellow vegetables, and, conversely, as reflecting stress-induced increases in the consumption of bread, noodles, and potatoes. In our food classification, bread, noodles, and potatoes are grouped as starchy foods; however, rice is not included in the analysis because the response categories for intake frequency in the *FFQ* differ from those used for other food items. Since bread, noodles, and potatoes are substitutes for rice in Japan, it should be noted that increases in the intake frequencies of these foods may be accompanied by decreases in rice consumption. Therefore, it is also possible that the observed positive association for starchy foods excluding rice reflects a decrease in the share of rice within the starchy-foods portfolio that is positively correlated with stress responses.

A particularly noteworthy implication of the present analysis is that it distinguishes whether these quantitative relationships are linear or nonlinear. The shape of the dose–response curve allows us to distinguish whether a given food group is one for which “the more, the better” or one for which “even a small amount is beneficial as long as it is consumed at all.”

Several limitations of this study must be acknowledged. First, because the present analysis is based on a cross-sectional study, it does not estimate temporal relationships or causality. Even after controlling for confounding factors, the possibility of reverse causation remains. Nevertheless, examining the relationship between diet and mental health—two domains that are mutually interdependent—through dose–response curves offers meaningful insights, particularly in distinguishing linear from nonlinear associations. From the perspective of dietary improvement, such distinctions help clarify whether consuming more of a given food is beneficial or whether even small amounts are sufficient. From the perspective of stress-response outcomes, this approach allows for a flexible characterization of how stress may alter dietary patterns. Second, there are issues related to the accuracy of *FFQ* data. Because FFQs rely on respondents’ memory, they are subject to recall bias, social desirability bias, and misclassification ([32] and its references). Even so, to the extent that these sources of measurement error arise primarily at the food-group level, the nature of the present modeling approach implies that such disturbances are absorbed into $\lambda_{k}^{\left(r\right)}$, thereby offering some degree of mitigation regarding their influence on the estimation of $\lambda_{i}^{\left(r\right)}$. Third, there are limitations concerning the coverage of the *FFQ*. In the *FFQ* used for this study, certain items—such as rice, a traditional staple food in Japan, and beverages including alcoholic drinks—were excluded from the analysis because their response categories differed from those of other items. Moreover, ultra-processed foods, restaurant meals, and dietary supplements were not included in the survey. The relationships between these foods and mental health are also non-negligible [28,33]. Other limitations include the large credibility intervals resulting from the small sample size, the fact that the sample is restricted to a single region in Hokkaido, potential sample bias inherent in cross-sectional data, and the fact that although the validity of the stress-response items in the Brief Job Stress Questionnaire has been examined using samples of employed individuals [21], our analysis is not restricted to employed respondents.

## 5. Conclusions

This study analyzed the quantitative relationships between food-group-specific intake and stress responses using observational data collected from adolescents and young adults in the area surrounding Ebetsu City, Hokkaido. To estimate dose–response relationships from *FFQ* data, which provide limited informational granularity, we constructed a structural equation model incorporating correlated error terms and generated posterior predictions through Bayesian estimation. The results indicated that dark-green and yellow vegetables exhibited a nonlinear negative association with stress responses, whereas bread, noodles, and potatoes showed a linear positive association. In other words, green and yellow vegetables show differences in stress responses depending on whether they are consumed or not, whereas bread, noodles, and potatoes (starchy foods other than rice) exhibit variations in stress responses according to their intake frequency.

## Data Availability

The data obtained from the “MyLife Health Survey” are not currently in a publicly accessible repository but are scheduled to be released in 2028 from a publicly accessible repository managed by the DDBJ (DNA Data Bank of Japan) and JGA (Japanese Genotype-phenotype Archive; https://www.ddbj.nig.ac.jp/jga/index-e.html accessed on 5 August 2026).

## Supplementary Materials

The following supporting information can be downloaded at: https://www.mdpi.com/article/10.3390/nu18162612/s1, Table S1: Comparison of complete-case and missing-data case samples; Table S2: Frequency distribution of stress response scale; Table S3: Frequency distribution of *FFQ* data; Table S4. Estimation and convergence diagnostics.

## Abbreviations

The following abbreviations are used in this manuscript:

AYA	Adolescents and young adults
*FFQ*	Food frequency questionnaire
HDI	Highest density interval
MCMC	Markov chain Monte Carlo
SEM	Structural equation model

## Appendix B

**Table A1:** Food categorization.

(a) Meat	Pacific saury, mackerel	Bean sprout
Beef steak	Shirasuboshi	Lettuce
Beef grilled	Cod roe, salmon roe	Garlic
Beef saute	Eel	Pickled radish
Beef stewed	Squid	Pickled plum
Pork saute	Octopus	Pickled Chinese cabbage
Pork fried	Shrimp	Pickled cucumber
Pork stewed	Clam, corb shell	Pickled eggplant
Pork simmered	(f) Fruit	Pickled turnip
Pork soup	Mandarin	Konjac
Pork liver	Other oranges	Mushroom (shitake)
Chicken grilled	Apple	Mushroom (enokitake)
Chicken saute	Persimmon	Mushroom (shimeji)
Chicken simmered	Strawberry	Seaweed (wakame)
Chicken fried	Grapes	Seaweed (hijiki)
Chicken liver	Melon	Dried seaweed (nori)
(b) Processed meat and fish products	Watermelon	(i) Bread, noodles, and potatoes
Ham	Peach	Bread
Sausage	Pears	Japanese noodles (udon)
Bacon	Kiwi fruit	Japanese noodles (soba)
Fish sausage	Pineapple	Japanese noodles (soumen)
Boiled fish paste	Banana	Chinese noodles
Fried fish paste	Jam, marmalade	Pasta
(c) Dairy products and eggs	(g) Dark-green and yellow vegetables	Rice cake
Low fat milk	Carrot	Sweet potatoes
Milk	Spinach	Potatoes
Cheeses	Pumpkin	Taros
Yogurt	Green pepper	Yams
Egg	Tomatoes	(j) Confectionary, seasonings, etc.
(d) Soy products	Green onion	Butter
Tofu in miso soup	Green chive	Margarine
Boiled tofu	Crown daisy	Salad dressings
Bean curd	Japanese mustard spinach	Mayonnaise
Fried bean curd	Broccoli	Worcester sauce
Deep-fried bean curd	Snap bean	Ketchup
Fermented soybeans	Green asparagus	Mustard
(e) Seafood	Pickled green vegetables	Wasabi
Salted fish	(h) Other vegetables, mushrooms, seaweeds, and konjac	Japanese cake
Dried fish	Cabbage	Cakes
Canned tuna	Radish	Biscuit, cookie
Salmon, trout	Japanese long onion	Chocolate
Tunas, bonito	Onion	Ice cream
Amberjack	Cucumber	Snacks
Pollack, flatfish	Eggplant	Rice cracker
Sea breams	Chinese cabbage	Sesame
Horse mackerel, sardine	Burdock	Peanuts

## Appendix C

**Table A2:** Descriptive statistics of basic attributes by gender in the analytical sample.

	Males (*n* = 48)				Females (*n* = 124)				*p*-Value ^8^
	Mean	Std	Min	Max	Mean	Std	Min	Max	
Age ^1^	32.3	5.23	19	39	29.8	5.77	18	39	0.007
BMI ^2^	22.6	3.62	16.5	37.7	21.2	3.67	16.1	37.5	0.026
University ^3^	0.729	0.449	0	1	0.540	0.500	0	1	0.019
Cohabitants ^4^	0.771	0.425	0	1	0.774	0.420	0	1	0.963
Employment ^5^	0.883	0.377	0	1	0.710	0.456	0	1	0.072
Activity L ^6^	0.271	0.449	0	1	0.258	0.439	0	1	0.867
Activity H ^7^	0.333	0.476	0	1	0.194	0.397	0	1	0.075

^1^ Age: years; ^2^ BMI: kg/m^2^; ^3^ University: university graduate; ^4^ Cohabitants: living with others/cohabiting; ^5^ Employment: employed; ^6^ Activity L: low physical activity level; ^7^ Activity H: high physical activity level; ^8^ the *p*-value from Welch’s *t*-test.

## Appendix D

**Table A3:** The proportion of increasing or decreasing posterior predictive dose–response relationships, and the degree of curvature of the curve.

		Males					Females				
		Increase ^1^	Decrease ^1^	Bend ^2^			Increase ^1^	Decrease ^1^	Bend ^2^		
				Mean	90% HDI				Mean	90% HDI	
Vigor	(a) Meat	0.76	0.24	0.61	0.46	0.77	0.80	0.20	0.59	0.43	0.75
	(b) Processed meat and fish products	0.72	0.28	0.66	0.51	0.82	0.92	0.08	0.60	0.47	0.73
	(c) Dairy products and eggs	0.25	0.75	0.78	0.67	0.89	0.42	0.58	0.75	0.63	0.86
	(d) Soy products	0.77	0.23	0.65	0.51	0.82	0.95	0.05	0.59	0.47	0.71
	(e) Seafood	0.56	0.44	0.66	0.51	0.82	0.42	0.58	0.69	0.53	0.84
	(f) Fruit	0.55	0.45	0.70	0.55	0.83	0.27	0.73	0.75	0.64	0.88
	(g) Dark-green and yellow vegetables	0.08	0.92	0.83	0.74	0.90	0.19	0.81	0.80	0.68	0.89
	(h) Other vegetables, mushrooms, etc.	0.23	0.77	0.79	0.65	0.89	0.43	0.57	0.74	0.58	0.89
	(i) Bread, noodles, and potatoes	0.88	0.12	0.57	0.42	0.73	0.95	0.05	0.53	0.40	0.68
	(j) Confectionery and seasonings, etc.	0.40	0.60	0.72	0.58	0.86	0.19	0.81	0.77	0.66	0.88
Anger	(a) Meat	0.72	0.28	0.62	0.45	0.79	0.28	0.72	0.73	0.59	0.87
	(b) Processed meat and fish products	0.36	0.64	0.75	0.60	0.88	0.52	0.48	0.71	0.56	0.88
	(c) Dairy products and eggs	0.82	0.18	0.64	0.49	0.79	0.54	0.46	0.72	0.60	0.86
	(d) Soy products	0.58	0.42	0.69	0.53	0.87	0.33	0.67	0.76	0.61	0.89
	(e) Seafood	0.61	0.39	0.65	0.48	0.82	0.50	0.50	0.67	0.50	0.86
	(f) Fruit	0.43	0.57	0.72	0.57	0.86	0.24	0.76	0.77	0.65	0.89
	(g) Dark-green and yellow vegetables	0.50	0.50	0.71	0.54	0.88	0.43	0.57	0.73	0.57	0.89
	(h) Other vegetables, mushrooms, etc.	0.62	0.38	0.68	0.50	0.86	0.45	0.55	0.73	0.56	0.89
	(i) Bread, noodles, and potatoes	0.49	0.52	0.69	0.52	0.86	0.80	0.20	0.59	0.41	0.78
	(j) Confectionery and seasonings, etc.	0.56	0.44	0.68	0.51	0.85	0.35	0.65	0.73	0.59	0.87
Fatigue	(a) Meat	0.67	0.33	0.65	0.54	0.77	0.25	0.75	0.72	0.62	0.82
	(b) Processed meat and fish products	0.30	0.70	0.76	0.67	0.86	0.43	0.57	0.73	0.63	0.84
	(c) Dairy products and eggs	0.44	0.56	0.75	0.66	0.85	0.70	0.30	0.72	0.63	0.80
	(d) Soy products	0.37	0.63	0.75	0.65	0.86	0.27	0.73	0.77	0.67	0.87
	(e) Seafood	0.73	0.27	0.64	0.51	0.75	0.81	0.19	0.61	0.47	0.75
	(f) Fruit	0.79	0.21	0.67	0.56	0.76	0.50	0.50	0.71	0.61	0.82
	(g) Dark-green and yellow vegetables	0.36	0.64	0.75	0.66	0.86	0.08	0.92	0.81	0.73	0.88
	(h) Other vegetables, mushrooms, etc.	0.35	0.65	0.76	0.65	0.86	0.19	0.81	0.78	0.69	0.87
	(i) Bread, noodles, and potatoes	0.83	0.17	0.63	0.51	0.74	0.98	0.02	0.57	0.47	0.67
	(j) Confectionery and seasonings, etc.	0.52	0.48	0.70	0.59	0.80	0.56	0.44	0.69	0.59	0.80
Anxiety	(a) Meat	0.43	0.57	0.69	0.56	0.83	0.27	0.73	0.73	0.59	0.86
	(b) Processed meat and fish products	0.40	0.60	0.74	0.60	0.87	0.51	0.49	0.71	0.57	0.86
	(c) Dairy products and eggs	0.16	0.84	0.81	0.71	0.89	0.05	0.96	0.84	0.77	0.90
	(d) Soy products	0.40	0.60	0.74	0.61	0.89	0.52	0.48	0.71	0.56	0.88
	(e) Seafood	0.76	0.24	0.61	0.46	0.78	0.67	0.33	0.63	0.45	0.82
	(f) Fruit	0.77	0.23	0.64	0.49	0.78	0.82	0.18	0.62	0.46	0.77
	(g) Dark-green and yellow vegetables	0.31	0.69	0.76	0.63	0.89	0.35	0.65	0.76	0.61	0.89
	(h) Other vegetables, mushrooms, etc.	0.41	0.59	0.74	0.59	0.88	0.22	0.78	0.79	0.65	0.89
	(i) Bread, noodles, and potatoes	0.74	0.26	0.62	0.46	0.79	0.98	0.02	0.49	0.38	0.61
	(j) Confectionery and seasonings, etc.	0.46	0.54	0.71	0.55	0.84	0.77	0.23	0.62	0.47	0.79
Depression	(a) Meat	0.72	0.28	0.62	0.45	0.77	0.43	0.57	0.69	0.54	0.85
	(b) Processed meat and fish products	0.55	0.45	0.70	0.54	0.86	0.93	0.07	0.58	0.45	0.71
	(c) Dairy products and eggs	0.39	0.61	0.76	0.62	0.89	0.67	0.33	0.70	0.57	0.83
	(d) Soy products	0.36	0.64	0.75	0.60	0.88	0.39	0.61	0.74	0.60	0.89
	(e) Seafood	0.71	0.29	0.62	0.45	0.79	0.78	0.22	0.59	0.39	0.77
	(f) Fruit	0.53	0.47	0.70	0.54	0.84	0.22	0.78	0.77	0.65	0.88
	(g) Dark-green and yellow vegetables	0.05	0.95	0.84	0.77	0.90	0.01	0.99	0.87	0.84	0.90
	(h) Other vegetables, mushrooms, etc.	0.64	0.36	0.68	0.51	0.85	0.75	0.25	0.64	0.48	0.83
	(i) Bread, noodles, and potatoes	0.88	0.12	0.56	0.41	0.74	1.00	0.00	0.44	0.35	0.52
	(j) Confectionery and seasonings, etc.	0.53	0.47	0.69	0.53	0.85	0.90	0.10	0.57	0.42	0.73
Physical	(a) Meat	0.20	0.80	0.75	0.63	0.87	0.34	0.66	0.72	0.56	0.87
	(b) Processed meat and fish products	0.01	0.99	0.87	0.82	0.90	0.01	0.99	0.87	0.82	0.90
	(c) Dairy products and eggs	0.73	0.27	0.67	0.53	0.84	0.06	0.94	0.83	0.76	0.90
	(d) Soy products	0.70	0.30	0.66	0.47	0.84	0.49	0.51	0.71	0.55	0.89
	(e) Seafood	0.64	0.36	0.64	0.46	0.81	0.58	0.42	0.65	0.46	0.84
	(f) Fruit	0.33	0.67	0.74	0.60	0.87	0.36	0.64	0.74	0.59	0.88
	(g) Dark-green and yellow vegetables	0.71	0.29	0.65	0.48	0.84	0.31	0.69	0.77	0.61	0.90
	(h) Other vegetables, mushrooms, etc.	0.64	0.36	0.68	0.50	0.86	0.66	0.34	0.67	0.48	0.87
	(i) Bread, noodles, and potatoes	0.86	0.14	0.56	0.38	0.74	0.98	0.02	0.46	0.33	0.60
	(j) Confectionery and seasonings, etc.	0.60	0.40	0.67	0.50	0.85	0.65	0.35	0.65	0.48	0.83

^1^ Increase and Decrease respectively represent the proportion of posterior predictive samples in which *P*(*Y*∣*λ*) is a strictly increasing function of *λ* and a strictly decreasing function of *λ*; ^2^ Bend is defined in Equation (8), and Mean and HDI denote the mean and the 90% HDI of the posterior predictive distribution of Bend.
