# Supplementary material for: Estimated Dose-Dependent Associations Between Dietary Intake and Stress Responses in Adolescents and Young Adults: Evidence from a Regional Observational Study in Japan
Source: Nutrients. 2026 Aug 10;18(16):2612. doi: 10.3390/nu18162612 (PMC13516492; doi:10.3390/nu18162612)
Supplement: Supplementary file 1 [file nutrients-18-02612-s001.zip › nutrients-4401701-supplementary.pdf]

Supplementary Materials

Table S1. Comparison of complete-case and missing-data case samples

	Complete-cases (n=172)				Missing-data cases (n=30)				p-value
	Mean	Std	Min	Max	Mean	Std	Min	Max	
Age	30.5	5.73	18	39	30.6	6.53	19	39	0.934
Female	0.72	0.45	0	1	0.67	0.48	0	1	0.698
Cohabitants	0.77	0.42	0	1	0.63	0.49	0	1	0.159
Employment	0.74	0.44	0	1	0.77	0.43	0	1	0.973
university	0.59	0.49	0	1	0.53	0.51	0	1	0.681

Note. Age: years; BMI: kg/m^2; University: university graduate; Cohabitants: living with others / cohabiting; Employment: employed. For age, the p-value is based on Welch's t-test, and for the other binary variables, it is based on the chi-square test.

Table S2. Frequency distribution of stress response scale

Vigor

	Low	Somewhat low	Moderate	Somewhat high	High	All	Low	Somewhat low	Moderate	Somewhat high	High
Males	3	6	21	12	6	48	6.3%	12.5%	43.8%	25.0%	12.5%
Females	9	13	54	29	19	124	7.3%	10.5%	43.5%	23.4%	15.3%
All	12	19	75	41	25	172	7.0%	11.0%	43.6%	23.8%	14.5%
P(chi2)	0.982										

Irritability

	Low	Somewhat low	Moderate	Somewhat high	High	All	Low	Somewhat low	Moderate	Somewhat high	High
Males	15	10	14	8	1	48	31.3%	20.8%	29.2%	16.7%	2.1%
Females	16	26	65	12	5	124	12.9%	21.0%	52.4%	9.7%	4.0%
All	31	36	79	20	6	172	18.0%	20.9%	45.9%	11.6%	3.5%
P(chi2)	0.015										

Fatigue

	Low	Somewhat low	Moderate	Somewhat high	High	All	Low	Somewhat low	Moderate	Somewhat high	High
Males	5	2	27	10	4	48	10.4%	4.2%	56.3%	20.8%	8.3%
Females	7	31	44	34	8	124	5.6%	25.0%	35.5%	27.4%	6.5%
All	12	33	71	44	12	172	7.0%	19.2%	41.3%	25.6%	7.0%
P(chi2)	0.010										

Anxiety

	Low	Somewhat low	Moderate	Somewhat high	High	All	Low	Somewhat low	Moderate	Somewhat high	High
Males	15	7	21	4	1	48	31.3%	14.6%	43.8%	8.3%	2.1%
Females	32	14	49	24	5	124	25.8%	11.3%	39.5%	19.4%	4.0%
All	47	21	70	28	6	172	27.3%	12.2%	40.7%	16.3%	3.5%
P(chi2)	0.435										

Depression

	Low	Somewhat low	Moderate	Somewhat high	High	All	Low	Somewhat low	Moderate	Somewhat high	High
Males	10	14	19	4	1	48	20.8%	29.2%	39.6%	8.3%	2.1%
Females	17	31	48	23	5	124	13.7%	25.0%	38.7%	18.5%	4.0%
All	27	45	67	27	6	172	15.7%	26.2%	39.0%	15.7%	3.5%
P(chi2)	0.402										

Physical complaint

	Low	Somewhat low	Moderate	Somewhat high	High	All	Low	Somewhat low	Moderate	Somewhat high	High
Males	3	10	27	7	1	48	6.3%	20.8%	56.3%	14.6%	2.1%
Females	21	36	52	14	1	124	16.9%	29.0%	41.9%	11.3%	0.8%
All	24	46	79	21	2	172	14.0%	26.7%	45.9%	12.2%	1.2%
P(chi2)	0.196										

Note. $P(chi^2)$ represents the p-value of the chi-square test assessing the independence between the stress response and gender.

Table S3. Frequency distribution of FFQ data

	Males						Females						P(chi2)
	< 1/month	1~3/month	1~2/week	3~4/week	5~6/week	≥1/day	< 1/month	1~3/month	1~2/week	3~4/week	5~6/week	≥1/day	
Beef steak	40 (83.3%)	7 (14.6%)	1 (2.1%)	0 (0%)	0 (0%)	0 (0%)	106 (85.5%)	16 (12.9%)	2 (1.6%)	0 (0%)	0 (0%)	0 (0%)	0.934
Beef grilled	10 (20.8%)	27 (56.3%)	8 (16.7%)	3 (6.3%)	0 (0%)	0 (0%)	49 (39.5%)	62 (50%)	11 (8.9%)	2 (1.6%)	0 (0%)	0 (0%)	0.041
Beef saute	16 (33.3%)	16 (33.3%)	13 (27.1%)	3 (6.3%)	0 (0%)	0 (0%)	70 (56.5%)	27 (21.8%)	24 (19.4%)	3 (2.4%)	0 (0%)	0 (0%)	0.047
Beef stewed	18 (37.5%)	19 (39.6%)	9 (18.8%)	1 (2.1%)	1 (2.1%)	0 (0%)	71 (57.3%)	41 (33.1%)	11 (8.9%)	1 (0.8%)	0 (0%)	0 (0%)	0.061
Pork saute	2 (4.2%)	16 (33.3%)	22 (45.8%)	8 (16.7%)	0 (0%)	0 (0%)	14 (11.3%)	34 (27.4%)	52 (41.9%)	21 (16.9%)	2 (1.6%)	1 (0.8%)	0.610
Pork fried	18 (37.5%)	19 (39.6%)	9 (18.8%)	2 (4.2%)	0 (0%)	0 (0%)	53 (42.7%)	56 (45.2%)	15 (12.1%)	0 (0%)	0 (0%)	0 (0%)	0.081
Pork stewed	14 (29.2%)	25 (52.1%)	6 (12.5%)	2 (4.2%)	1 (2.1%)	0 (0%)	40 (32.3%)	62 (50%)	19 (15.3%)	3 (2.4%)	0 (0%)	0 (0%)	0.513
Pork simmered	34 (70.8%)	12 (25%)	2 (4.2%)	0 (0%)	0 (0%)	0 (0%)	97 (78.2%)	21 (16.9%)	5 (4%)	1 (0.8%)	0 (0%)	0 (0%)	0.613
Pork soup	27 (56.3%)	19 (39.6%)	1 (2.1%)	0 (0%)	0 (0%)	1 (2.1%)	69 (55.6%)	48 (38.7%)	7 (5.6%)	0 (0%)	0 (0%)	0 (0%)	0.316
Pork liver	38 (79.2%)	9 (18.8%)	1 (2.1%)	0 (0%)	0 (0%)	0 (0%)	108 (87.1%)	16 (12.9%)	0 (0%)	0 (0%)	0 (0%)	0 (0%)	0.161
Chicken grilled	12 (25%)	24 (50%)	7 (14.6%)	5 (10.4%)	0 (0%)	0 (0%)	23 (18.5%)	61 (49.2%)	34 (27.4%)	6 (4.8%)	0 (0%)	0 (0%)	0.188
Chicken saute	11 (22.9%)	18 (37.5%)	15 (31.3%)	4 (8.3%)	0 (0%)	0 (0%)	41 (33.1%)	39 (31.5%)	38 (30.6%)	6 (4.8%)	0 (0%)	0 (0%)	0.514
Chicken simmered	25 (52.1%)	19 (39.6%)	4 (8.3%)	0 (0%)	0 (0%)	0 (0%)	62 (50%)	43 (34.7%)	19 (15.3%)	0 (0%)	0 (0%)	0 (0%)	0.467
Chicken fried	9 (18.8%)	24 (50%)	7 (14.6%)	5 (10.4%)	3 (6.3%)	0 (0%)	20 (16.1%)	68 (54.8%)	33 (26.6%)	3 (2.4%)	0 (0%)	0 (0%)	0.005
Chicken liver	41 (85.4%)	7 (14.6%)	0 (0%)	0 (0%)	0 (0%)	0 (0%)	111 (89.5%)	13 (10.5%)	0 (0%)	0 (0%)	0 (0%)	0 (0%)	0.626
Ham	27 (56.3%)	14 (29.2%)	3 (6.3%)	3 (6.3%)	0 (0%)	1 (2.1%)	60 (48.4%)	43 (34.7%)	14 (11.3%)	6 (4.8%)	1 (0.8%)	0 (0%)	0.449
Sausage	11 (22.9%)	16 (33.3%)	9 (18.8%)	7 (14.6%)	3 (6.3%)	2 (4.2%)	28 (22.6%)	54 (43.5%)	26 (21%)	12 (9.7%)	3 (2.4%)	1 (0.8%)	0.367
Bacon	16 (33.3%)	26 (54.2%)	5 (10.4%)	1 (2.1%)	0 (0%)	0 (0%)	51 (41.1%)	52 (41.9%)	16 (12.9%)	5 (4%)	0 (0%)	0 (0%)	0.525
Low fat milk	29 (60.4%)	5 (10.4%)	4 (8.3%)	5 (10.4%)	1 (2.1%)	4 (8.3%)	89 (71.8%)	12 (9.7%)	6 (4.8%)	6 (4.8%)	5 (4%)	6 (4.8%)	0.635
Milk	24 (50%)	11 (22.9%)	6 (12.5%)	3 (6.3%)	3 (6.3%)	1 (2.1%)	47 (37.9%)	21 (16.9%)	18 (14.5%)	12 (9.7%)	10 (8.1%)	16 (12.9%)	0.333
Egg	0 (0%)	1 (2.1%)	8 (16.7%)	21 (43.8%)	10 (20.8%)	8 (16.7%)	1 (0.8%)	9 (7.3%)	37 (29.8%)	37 (29.8%)	21 (16.9%)	19 (15.3%)	0.317
Cheeses	10 (20.8%)	16 (33.3%)	18 (37.5%)	3 (6.3%)	0 (0%)	1 (2.1%)	17 (13.7%)	46 (37.1%)	35 (28.2%)	18 (14.5%)	5 (4%)	3 (2.4%)	0.296
Yogurt	9 (18.8%)	16 (33.3%)	4 (8.3%)	12 (25%)	0 (0%)	7 (14.6%)	26 (21%)	26 (21%)	29 (23.4%)	20 (16.1%)	10 (8.1%)	13 (10.5%)	0.032
Salted fish	19 (39.6%)	25 (52.1%)	2 (4.2%)	1 (2.1%)	0 (0%)	1 (2.1%)	45 (36.3%)	63 (50.8%)	16 (12.9%)	0 (0%)	0 (0%)	0 (0%)	0.099
Dried fish	33 (68.8%)	13 (27.1%)	0 (0%)	2 (4.2%)	0 (0%)	0 (0%)	88 (71%)	31 (25%)	5 (4%)	0 (0%)	0 (0%)	0 (0%)	0.066
Canned tuna	23 (47.9%)	16 (33.3%)	6 (12.5%)	2 (4.2%)	1 (2.1%)	0 (0%)	55 (44.4%)	46 (37.1%)	21 (16.9%)	2 (1.6%)	0 (0%)	0 (0%)	0.377
Salmon, trout	20 (41.7%)	21 (43.8%)	4 (8.3%)	2 (4.2%)	1 (2.1%)	0 (0%)	45 (36.3%)	64 (51.6%)	15 (12.1%)	0 (0%)	0 (0%)	0 (0%)	0.064
Tunas, bonito	17 (35.4%)	25 (52.1%)	5 (10.4%)	1 (2.1%)	0 (0%)	0 (0%)	55 (44.4%)	62 (50%)	7 (5.6%)	0 (0%)	0 (0%)	0 (0%)	0.221
Amberjack	25 (52.1%)	21 (43.8%)	2 (4.2%)	0 (0%)	0 (0%)	0 (0%)	86 (69.4%)	32 (25.8%)	6 (4.8%)	0 (0%)	0 (0%)	0 (0%)	0.072
Pollack, flatfish	33 (68.8%)	13 (27.1%)	2 (4.2%)	0 (0%)	0 (0%)	0 (0%)	93 (75%)	30 (24.2%)	1 (0.8%)	0 (0%)	0 (0%)	0 (0%)	0.281
Sea breams	40 (83.3%)	7 (14.6%)	1 (2.1%)	0 (0%)	0 (0%)	0 (0%)	113 (91.1%)	11 (8.9%)	0 (0%)	0 (0%)	0 (0%)	0 (0%)	0.142
Horse mackerel, sardine	39 (81.3%)	9 (18.8%)	0 (0%)	0 (0%)	0 (0%)	0 (0%)	104 (83.9%)	17 (13.7%)	3 (2.4%)	0 (0%)	0 (0%)	0 (0%)	0.412
Pacific saury, mackerel	22 (45.8%)	23 (47.9%)	1 (2.1%)	2 (4.2%)	0 (0%)	0 (0%)	56 (45.2%)	54 (43.5%)	14 (11.3%)	0 (0%)	0 (0%)	0 (0%)	0.034
Shirasuboshi	39 (81.3%)	5 (10.4%)	3 (6.3%)	1 (2.1%)	0 (0%)	0 (0%)	86 (69.4%)	29 (23.4%)	8 (6.5%)	0 (0%)	1 (0.8%)	0 (0%)	0.159
Cod roe, salmon roe	30 (62.5%)	15 (31.3%)	2 (4.2%)	1 (2.1%)	0 (0%)	0 (0%)	68 (54.8%)	48 (38.7%)	4 (3.2%)	3 (2.4%)	1 (0.8%)	0 (0%)	0.849
Eel	45 (93.8%)	3 (6.3%)	0 (0%)	0 (0%)	0 (0%)	0 (0%)	117 (94.4%)	6 (4.8%)	1 (0.8%)	0 (0%)	0 (0%)	0 (0%)	0.771
Squid	32 (66.7%)	13 (27.1%)	3 (6.3%)	0 (0%)	0 (0%)	0 (0%)	85 (68.5%)	34 (27.4%)	5 (4%)	0 (0%)	0 (0%)	0 (0%)	0.825
Octopus	33 (68.8%)	13 (27.1%)	2 (4.2%)	0 (0%)	0 (0%)	0 (0%)	89 (71.8%)	35 (28.2%)	0 (0%)	0 (0%)	0 (0%)	0 (0%)	0.073
Shrimp	26 (54.2%)	21 (43.8%)	1 (2.1%)	0 (0%)	0 (0%)	0 (0%)	49 (39.5%)	60 (48.4%)	13 (10.5%)	1 (0.8%)	1 (0.8%)	0 (0%)	0.228
Clam, corb shell	38 (79.2%)	9 (18.8%)	1 (2.1%)	0 (0%)	0 (0%)	0 (0%)	85 (68.5%)	34 (27.4%)	5 (4%)	0 (0%)	0 (0%)	0 (0%)	0.375
Fish sausage (chikuwa)	26 (54.2%)	15 (31.3%)	4 (8.3%)	1 (2.1%)	2 (4.2%)	0 (0%)	63 (50.8%)	38 (30.6%)	17 (13.7%)	5 (4%)	0 (0%)	1 (0.8%)	0.234
Boiled fish paste (kamaboko)	40 (83.3%)	6 (12.5%)	2 (4.2%)	0 (0%)	0 (0%)	0 (0%)	100 (80.6%)	18 (14.5%)	4 (3.2%)	2 (1.6%)	0 (0%)	0 (0%)	0.803
Fried fish paste (satsuma-age)	41 (85.4%)	4 (8.3%)	3 (6.3%)	0 (0%)	0 (0%)	0 (0%)	93 (75%)	28 (22.6%)	2 (1.6%)	1 (0.8%)	0 (0%)	0 (0%)	0.066
Mandarin	22 (45.8%)	10 (20.8%)	7 (14.6%)	8 (16.7%)	1 (2.1%)	0 (0%)	43 (34.7%)	35 (28.2%)	23 (18.5%)	16 (12.9%)	2 (1.6%)	5 (4%)	0.609
Other oranges	32 (66.7%)	9 (18.8%)	6 (12.5%)	1 (2.1%)	0 (0%)	0 (0%)	72 (58.1%)	38 (30.6%)	9 (7.3%)	4 (3.2%)	0 (0%)	1 (0.8%)	0.427
Apple	26 (54.2%)	16 (33.3%)	2 (4.2%)	2 (4.2%)	2 (4.2%)	0 (0%)	60 (48.4%)	42 (33.9%)	16 (12.9%)	4 (3.2%)	2 (1.6%)	0 (0%)	0.431
Persimmon	39 (81.3%)	7 (14.6%)	1 (2.1%)	0 (0%)	1 (2.1%)	0 (0%)	74 (59.7%)	30 (24.2%)	18 (14.5%)	2 (1.6%)	0 (0%)	0 (0%)	0.016
Strawberry	28 (58.3%)	16 (33.3%)	4 (8.3%)	0 (0%)	0 (0%)	0 (0%)	49 (39.5%)	55 (44.4%)	16 (12.9%)	3 (2.4%)	1 (0.8%)	0 (0%)	0.205
Grapes	26 (54.2%)	16 (33.3%)	4 (8.3%)	1 (2.1%)	1 (2.1%)	0 (0%)	51 (41.1%)	51 (41.1%)	20 (16.1%)	2 (1.6%)	0 (0%)	0 (0%)	0.200
Melon	34 (70.8%)	12 (25%)	2 (4.2%)	0 (0%)	0 (0%)	0 (0%)	74 (59.7%)	37 (29.8%)	13 (10.5%)	0 (0%)	0 (0%)	0 (0%)	0.279
Watermelon	32 (66.7%)	12 (25%)	3 (6.3%)	0 (0%)	1 (2.1%)	0 (0%)	60 (48.4%)	46 (37.1%)	14 (11.3%)	3 (2.4%)	0 (0%)	1 (0.8%)	0.122
Peach	34 (70.8%)	9 (18.8%)	5 (10.4%)	0 (0%)	0 (0%)	0 (0%)	61 (49.2%)	49 (39.5%)	12 (9.7%)	2 (1.6%)	0 (0%)	0 (0%)	0.043
Pears	34 (70.8%)	9 (18.8%)	4 (8.3%)	1 (2.1%)	0 (0%)	0 (0%)	70 (56.5%)	41 (33.1%)	11 (8.9%)	2 (1.6%)	0 (0%)	0 (0%)	0.298
Kiwi fruit	28 (58.3%)	14 (29.2%)	4 (8.3%)	2 (4.2%)	0 (0%)	0 (0%)	61 (49.2%)	29 (23.4%)	22 (17.7%)	10 (8.1%)	2 (1.6%)	0 (0%)	0.334
Pineapple	35 (72.9%)	9 (18.8%)	2 (4.2%)	1 (2.1%)	1 (2.1%)	0 (0%)	75 (60.5%)	33 (26.6%)	14 (11.3%)	2 (1.6%)	0 (0%)	0 (0%)	0.183
Banana	18 (37.5%)	11 (22.9%)	10 (20.8%)	6 (12.5%)	1 (2.1%)	2 (4.2%)	41 (33.1%)	38 (30.6%)	23 (18.5%)	14 (11.3%)	4 (3.2%)	4 (3.2%)	0.934
Pickled radish	28 (58.3%)	11 (22.9%)	5 (10.4%)	3 (6.3%)	1 (2.1%)	0 (0%)	94 (75.8%)	25 (20.2%)	5 (4%)	0 (0%)	0 (0%)	0 (0%)	0.006
Pickled green vegetables	38 (79.2%)	8 (16.7%)	1 (2.1%)	1 (2.1%)	0 (0%)	0 (0%)	104 (83.9%)	17 (13.7%)	3 (2.4%)	0 (0%)	0 (0%)	0 (0%)	0.407
Pickled plum	32 (66.7%)	8 (16.7%)	4 (8.3%)	3 (6.3%)	1 (2.1%)	0 (0%)	65 (52.4%)	40 (32.3%)	13 (10.5%)	4 (3.2%)	1 (0.8%)	1 (0.8%)	0.299
Pickled Chinese cabbage	35 (72.9%)	10 (20.8%)	3 (6.3%)	0 (0%)	0 (0%)	0 (0%)	94 (75.8%)	22 (17.7%)	6 (4.8%)	1 (0.8%)	1 (0.8%)	0 (0%)	0.891
Pickled cucumber	26 (54.2%)	12 (25%)	4 (8.3%)	4 (8.3%)	1 (2.1%)	1 (2.1%)	61 (49.2%)	34 (27.4%)	20 (16.1%)	7 (5.6%)	2 (1.6%)	0 (0%)	0.445
Pickled eggplant	41 (85.4%)	4 (8.3%)	3 (6.3%)	0 (0%)	0 (0%)	0 (0%)	108 (87.1%)	11 (8.9%)	4 (3.2%)	1 (0.8%)	0 (0%)	0 (0%)	0.756
Pickled turnip	41 (85.4%)	6 (12.5%)	0 (0%)	0 (0%)	1 (2.1%)	0 (0%)	104 (83.9%)	19 (15.3%)	1 (0.8%)	0 (0%)	0 (0%)	0 (0%)	0.366

Note. The "≥1/day" category was aggregated from four original categories: "1/day", "2–3/day", "4–6/day", and "≥7/day". P(chi^2) represents the p-value of the chi-square test assessing the independence between the FFQ and gender.

Table S3 (Continued). Frequency distribution of FFQ data

	Males						Females						P(chi2)
	< 1/month	1~3/month	1~2/week	3~4/week	5~6/week	≥1/day	< 1/month	1~3/month	1~2/week	3~4/week	5~6/week	≥1/day	
Carrot	4 (8.3%)	15 (31.3%)	17 (35.4%)	7 (14.6%)	1 (2.1%)	4 (8.3%)	6 (4.8%)	35 (28.2%)	37 (29.8%)	34 (27.4%)	9 (7.3%)	3 (2.4%)	0.149
Spinach	7 (14.6%)	22 (45.8%)	16 (33.3%)	3 (6.3%)	0 (0%)	0 (0%)	24 (19.4%)	48 (38.7%)	39 (31.5%)	13 (10.5%)	0 (0%)	0 (0%)	0.665
Pumpkin	17 (35.4%)	24 (50%)	5 (10.4%)	1 (2.1%)	0 (0%)	1 (2.1%)	39 (31.5%)	52 (41.9%)	25 (20.2%)	7 (5.6%)	1 (0.8%)	0 (0%)	0.263
Cabbage	1 (2.1%)	13 (27.1%)	21 (43.8%)	8 (16.7%)	2 (4.2%)	3 (6.2%)	5 (4%)	23 (18.5%)	46 (37.1%)	36 (29%)	11 (8.9%)	3 (2.4%)	0.291
Radish	12 (25%)	19 (39.6%)	15 (31.3%)	1 (2.1%)	0 (0%)	1 (2.1%)	13 (10.5%)	44 (35.5%)	41 (33.1%)	21 (16.9%)	5 (4%)	0 (0%)	0.008
Green pepper	5 (10.4%)	21 (43.8%)	14 (29.2%)	6 (12.5%)	0 (0%)	2 (4.2%)	16 (12.9%)	46 (37.1%)	41 (33.1%)	16 (12.9%)	3 (2.4%)	2 (1.6%)	0.606
Tomatoes	5 (10.4%)	10 (20.8%)	16 (33.3%)	11 (22.9%)	3 (6.3%)	3 (6.3%)	19 (15.3%)	23 (18.5%)	34 (27.4%)	25 (20.2%)	13 (10.5%)	10 (8.1%)	0.798
Japanese long onion	4 (8.3%)	15 (31.3%)	17 (35.4%)	9 (18.8%)	2 (4.2%)	1 (2.1%)	16 (12.9%)	38 (30.6%)	36 (29%)	23 (18.5%)	7 (5.6%)	4 (3.2%)	0.950
Green onion	18 (37.5%)	11 (22.9%)	11 (22.9%)	7 (14.6%)	0 (0%)	1 (2.1%)	67 (54%)	30 (24.2%)	16 (12.9%)	6 (4.8%)	3 (2.4%)	2 (1.6%)	0.104
Green chive	13 (27.1%)	24 (50%)	8 (16.7%)	2 (4.2%)	0 (0%)	1 (2.1%)	45 (36.3%)	49 (39.5%)	24 (19.4%)	5 (4%)	1 (0.8%)	0 (0%)	0.432
Green vegetable (shungiku)	41 (85.4%)	6 (12.5%)	1 (2.1%)	0 (0%)	0 (0%)	0 (0%)	95 (76.6%)	23 (18.5%)	5 (4%)	1 (0.8%)	0 (0%)	0 (0%)	0.603
Green vegetable (komatsuna)	17 (35.4%)	17 (35.4%)	7 (14.6%)	6 (12.5%)	1 (2.1%)	0 (0%)	45 (36.3%)	48 (38.7%)	15 (12.1%)	14 (11.3%)	2 (1.6%)	0 (0%)	0.986
Broccoli	10 (20.8%)	18 (37.5%)	12 (25%)	5 (10.4%)	3 (6.3%)	0 (0%)	17 (13.7%)	34 (27.4%)	39 (31.5%)	22 (17.7%)	8 (6.5%)	4 (3.2%)	0.358
Onion	0 (0%)	7 (14.6%)	17 (35.4%)	13 (27.1%)	6 (12.5%)	5 (10.4%)	1 (0.8%)	18 (14.5%)	34 (27.4%)	38 (30.6%)	26 (21%)	7 (5.6%)	0.587
Cucumber	9 (18.8%)	11 (22.9%)	11 (22.9%)	11 (22.9%)	4 (8.3%)	2 (4.2%)	18 (14.5%)	21 (16.9%)	41 (33.1%)	23 (18.5%)	15 (12.1%)	6 (4.8%)	0.692
Eggplant	11 (22.9%)	13 (27.1%)	12 (25%)	10 (20.8%)	1 (2.1%)	1 (2.1%)	22 (17.7%)	34 (27.4%)	48 (38.7%)	16 (12.9%)	1 (0.8%)	3 (2.4%)	0.582
Chinese cabbage	8 (16.7%)	16 (33.3%)	18 (37.5%)	4 (8.3%)	1 (2.1%)	1 (2.1%)	20 (16.1%)	39 (31.5%)	33 (26.6%)	26 (21%)	4 (3.2%)	2 (1.6%)	0.230
Burdock	22 (45.8%)	17 (35.4%)	6 (12.5%)	2 (4.2%)	1 (2.1%)	0 (0%)	55 (44.4%)	44 (35.5%)	22 (17.7%)	3 (2.4%)	0 (0%)	0 (0%)	0.471
Bean sprout	4 (8.3%)	17 (35.4%)	12 (25%)	11 (22.9%)	2 (4.2%)	2 (4.2%)	17 (13.7%)	38 (30.6%)	46 (37.1%)	19 (15.3%)	2 (1.6%)	2 (1.6%)	0.293
Snap bean	36 (75%)	9 (18.8%)	3 (6.3%)	0 (0%)	0 (0%)	0 (0%)	95 (76.6%)	17 (13.7%)	8 (6.5%)	3 (2.4%)	1 (0.8%)	0 (0%)	0.709
Lettuce	13 (27.1%)	18 (37.5%)	8 (16.7%)	6 (12.5%)	2 (4.2%)	1 (2.1%)	20 (16.1%)	45 (36.3%)	31 (25%)	18 (14.5%)	7 (5.6%)	3 (2.4%)	0.577
Green asparagus	19 (39.6%)	19 (39.6%)	5 (10.4%)	5 (10.4%)	0 (0%)	0 (0%)	44 (35.5%)	47 (37.9%)	24 (19.4%)	7 (5.6%)	2 (1.6%)	0 (0%)	0.444
Garlic	20 (41.7%)	13 (27.1%)	9 (18.8%)	4 (8.3%)	0 (0%)	2 (4.2%)	50 (40.3%)	37 (29.8%)	19 (15.3%)	12 (9.7%)	6 (4.8%)	0 (0%)	0.245
Bread	2 (4.2%)	12 (25%)	15 (31.3%)	7 (14.6%)	3 (6.3%)	9 (18.8%)	5 (4%)	29 (23.4%)	47 (37.9%)	22 (17.7%)	12 (9.7%)	9 (7.3%)	0.423
Japanese noodles (udon)	3 (6.3%)	26 (54.2%)	17 (35.4%)	2 (4.2%)	0 (0%)	0 (0%)	25 (20.2%)	55 (44.4%)	39 (31.5%)	4 (3.2%)	1 (0.8%)	0 (0%)	0.243
Japanese noodles (soba)	8 (16.7%)	31 (64.6%)	6 (12.5%)	3 (6.3%)	0 (0%)	0 (0%)	49 (39.5%)	54 (43.5%)	20 (16.1%)	1 (0.8%)	0 (0%)	0 (0%)	0.004
Chinese noodles	4 (8.3%)	29 (60.4%)	14 (29.2%)	1 (2.1%)	0 (0%)	0 (0%)	29 (23.4%)	70 (56.5%)	20 (16.1%)	5 (4%)	0 (0%)	0 (0%)	0.057
Pasta	5 (10.4%)	29 (60.4%)	12 (25%)	1 (2.1%)	0 (0%)	1 (2.1%)	27 (21.8%)	73 (58.9%)	22 (17.7%)	1 (0.8%)	1 (0.8%)	0 (0%)	0.237
Japanese noodles (soumen)	21 (43.8%)	18 (37.5%)	8 (16.7%)	1 (2.1%)	0 (0%)	0 (0%)	53 (42.7%)	55 (44.4%)	14 (11.3%)	2 (1.6%)	0 (0%)	0 (0%)	0.749
Rice cake	36 (75%)	12 (25%)	0 (0%)	0 (0%)	0 (0%)	0 (0%)	100 (80.6%)	22 (17.7%)	2 (1.6%)	0 (0%)	0 (0%)	0 (0%)	0.399
Japanese cake	20 (41.7%)	19 (39.6%)	7 (14.6%)	1 (2.1%)	1 (2.1%)	0 (0%)	68 (54.8%)	46 (37.1%)	7 (5.6%)	3 (2.4%)	0 (0%)	0 (0%)	0.124
Cakes	23 (47.9%)	19 (39.6%)	6 (12.5%)	0 (0%)	0 (0%)	0 (0%)	64 (51.6%)	54 (43.5%)	4 (3.2%)	1 (0.8%)	0 (0%)	1 (0.8%)	0.191
Biscuit, cookie	17 (35.4%)	21 (43.8%)	8 (16.7%)	2 (4.2%)	0 (0%)	0 (0%)	29 (23.4%)	48 (38.7%)	32 (25.8%)	13 (10.5%)	1 (0.8%)	1 (0.8%)	0.337
Chocolate	11 (22.9%)	19 (39.6%)	10 (20.8%)	2 (4.2%)	3 (6.3%)	3 (6.2%)	17 (13.7%)	31 (25%)	39 (31.5%)	25 (20.2%)	9 (7.3%)	3 (2.4%)	0.017
Ice cream	5 (10.4%)	20 (41.7%)	15 (31.3%)	6 (12.5%)	2 (4.2%)	0 (0%)	17 (13.7%)	40 (32.3%)	43 (34.7%)	16 (12.9%)	5 (4%)	3 (2.4%)	0.783
Snacks	7 (14.6%)	20 (41.7%)	15 (31.3%)	3 (6.3%)	3 (6.3%)	0 (0%)	22 (17.7%)	42 (33.9%)	40 (32.3%)	18 (14.5%)	2 (1.6%)	0 (0%)	0.258
Rice cracker	25 (52.1%)	17 (35.4%)	6 (12.5%)	0 (0%)	0 (0%)	0 (0%)	57 (46%)	42 (33.9%)	21 (16.9%)	4 (3.2%)	0 (0%)	0 (0%)	0.517
Sesame	24 (50%)	15 (31.3%)	8 (16.7%)	1 (2.1%)	0 (0%)	0 (0%)	47 (37.9%)	33 (26.6%)	26 (21%)	12 (9.7%)	5 (4%)	1 (0.8%)	0.238
Peanuts	32 (66.7%)	11 (22.9%)	4 (8.3%)	1 (2.1%)	0 (0%)	0 (0%)	93 (75%)	20 (16.1%)	7 (5.6%)	2 (1.6%)	1 (0.8%)	1 (0.8%)	0.788
Tofu in miso soup	13 (27.1%)	12 (25%)	17 (35.4%)	3 (6.3%)	1 (2.1%)	2 (4.2%)	29 (23.4%)	41 (33.1%)	38 (30.6%)	11 (8.9%)	4 (3.2%)	1 (0.8%)	0.570
Boiled tofu	16 (33.3%)	17 (35.4%)	9 (18.8%)	4 (8.3%)	0 (0%)	2 (4.2%)	40 (32.3%)	45 (36.3%)	30 (24.2%)	5 (4%)	3 (2.4%)	1 (0.8%)	0.404
Bean curd (koya-tofu)	38 (79.2%)	7 (14.6%)	3 (6.3%)	0 (0%)	0 (0%)	0 (0%)	108 (87.1%)	14 (11.3%)	2 (1.6%)	0 (0%)	0 (0%)	0 (0%)	0.210
Fried bean curd	33 (68.8%)	12 (25%)	3 (6.3%)	0 (0%)	0 (0%)	0 (0%)	73 (58.9%)	42 (33.9%)	8 (6.5%)	1 (0.8%)	0 (0%)	0 (0%)	0.614
Deep-Fried bean curd	22 (45.8%)	17 (35.4%)	8 (16.7%)	1 (2.1%)	0 (0%)	0 (0%)	57 (46%)	46 (37.1%)	16 (12.9%)	2 (1.6%)	1 (0.8%)	2 (1.6%)	0.903
Fermented soybeans	4 (8.3%)	10 (20.8%)	19 (39.6%)	8 (16.7%)	1 (2.1%)	6 (12.5%)	25 (20.2%)	35 (28.2%)	43 (34.7%)	12 (9.7%)	3 (2.4%)	6 (4.8%)	0.145
Sweet potatoes	25 (52.1%)	20 (41.7%)	3 (6.3%)	0 (0%)	0 (0%)	0 (0%)	57 (46%)	40 (32.3%)	15 (12.1%)	8 (6.5%)	3 (2.4%)	1 (0.8%)	0.226
Potatoes	4 (8.3%)	21 (43.8%)	19 (39.6%)	3 (6.3%)	0 (0%)	1 (2.1%)	16 (12.9%)	50 (40.3%)	45 (36.3%)	11 (8.9%)	2 (1.6%)	0 (0%)	0.484
Taros	37 (77.1%)	9 (18.8%)	2 (4.2%)	0 (0%)	0 (0%)	0 (0%)	112 (90.3%)	12 (9.7%)	0 (0%)	0 (0%)	0 (0%)	0 (0%)	0.017
Yams	27 (56.3%)	16 (33.3%)	4 (8.3%)	1 (2.1%)	0 (0%)	0 (0%)	81 (65.3%)	34 (27.4%)	8 (6.5%)	1 (0.8%)	0 (0%)	0 (0%)	0.675
Konjac	26 (54.2%)	14 (29.2%)	8 (16.7%)	0 (0%)	0 (0%)	0 (0%)	57 (46%)	51 (41.1%)	15 (12.1%)	1 (0.8%)	0 (0%)	0 (0%)	0.437
Mushroom (shitake)	24 (50%)	18 (37.5%)	6 (12.5%)	0 (0%)	0 (0%)	0 (0%)	50 (40.3%)	47 (37.9%)	23 (18.5%)	3 (2.4%)	1 (0.8%)	0 (0%)	0.549
Mushroom (enokitake)	12 (25%)	22 (45.8%)	13 (27.1%)	1 (2.1%)	0 (0%)	0 (0%)	31 (25%)	46 (37.1%)	36 (29%)	10 (8.1%)	1 (0.8%)	0 (0%)	0.552
Mushroom (shimeji)	16 (33.3%)	17 (35.4%)	13 (27.1%)	1 (2.1%)	0 (0%)	1 (2.1%)	34 (27.4%)	41 (33.1%)	38 (30.6%)	9 (7.3%)	2 (1.6%)	0 (0%)	0.350
Seaweed (wakame)	9 (18.8%)	15 (31.3%)	19 (39.6%)	4 (8.3%)	0 (0%)	1 (2.1%)	23 (18.5%)	37 (29.8%)	43 (34.7%)	18 (14.5%)	3 (2.4%)	0 (0%)	0.412
Seaweed (hijiki)	27 (56.3%)	15 (31.3%)	5 (10.4%)	1 (2.1%)	0 (0%)	0 (0%)	80 (64.5%)	36 (29%)	6 (4.8%)	2 (1.6%)	0 (0%)	0 (0%)	0.539
Dried seaweed (nori)	14 (29.2%)	18 (37.5%)	13 (27.1%)	3 (6.3%)	0 (0%)	0 (0%)	36 (29%)	46 (37.1%)	23 (18.5%)	14 (11.3%)	4 (3.2%)	1 (0.8%)	0.545
Butter	33 (68.8%)	12 (25%)	1 (2.1%)	1 (2.1%)	0 (0%)	1 (2.1%)	78 (62.9%)	28 (22.6%)	16 (12.9%)	2 (1.6%)	0 (0%)	0 (0%)	0.136
Margarine	39 (81.3%)	7 (14.6%)	1 (2.1%)	0 (0%)	1 (2.1%)	0 (0%)	100 (80.6%)	14 (11.3%)	6 (4.8%)	2 (1.6%)	1 (0.8%)	1 (0.8%)	0.761
Jam, marmalade	32 (66.7%)	11 (22.9%)	3 (6.3%)	2 (4.2%)	0 (0%)	0 (0%)	86 (69.4%)	23 (18.5%)	12 (9.7%)	1 (0.8%)	1 (0.8%)	1 (0.8%)	0.571
Salad dressings	7 (14.6%)	20 (41.7%)	11 (22.9%)	6 (12.5%)	3 (6.3%)	1 (2.1%)	29 (23.4%)	33 (26.6%)	37 (29.8%)	14 (11.3%)	7 (5.6%)	4 (3.2%)	0.196
Mayonnaise	14 (29.2%)	13 (27.1%)	11 (22.9%)	7 (14.6%)	1 (2.1%)	2 (4.2%)	21 (16.9%)	42 (33.9%)	37 (29.8%)	20 (16.1%)	4 (3.2%)	0 (0%)	0.173
Worcester sauce	9 (18.8%)	20 (41.7%)	12 (25%)	6 (12.5%)	1 (2.1%)	0 (0%)	32 (25.8%)	55 (44.4%)	31 (25%)	4 (3.2%)	2 (1.6%)	0 (0%)	0.203
Ketchup	8 (16.7%)	21 (43.8%)	11 (22.9%)	6 (12.5%)	1 (2.1%)	1 (2.1%)	19 (15.3%)	58 (46.8%)	40 (32.3%)	5 (4%)	2 (1.6%)	0 (0%)	0.178
Mustard	22 (45.8%)	17 (35.4%)	4 (8.3%)	4 (8.3%)	0 (0%)	1 (2.1%)	98 (79%)	20 (16.1%)	3 (2.4%)	3 (2.4%)	0 (0%)	0 (0%)	0.001
Wasabi	13 (27.1%)	25 (52.1%)	6 (12.5%)	4 (8.3%)	0 (0%)	0 (0%)	64 (51.6%)	38 (30.6%)	16 (12.9%)	5 (4%)	1 (0.8%)	0 (0%)	0.031

Note. The "≥1/day" category was aggregated from four original categories: "1/day", "2–3/day", "4–6/day", and "≥7/day". P(chi²) represents the p-value of the chi-square test assessing the independence between the FFQ and gender.

Table S4. Estimation and convergence diagnostics

Regressand: Vigor

Regressor	Mean	HDI 3%	HDI 97%	MCSE Meran	ESS Bulk	R-hat
(a) Meat	0.468	-0.763	1.663	0.01	4404	1
(b) Processed meat and fish products	0.364	-0.856	1.505	0.011	3334	1
(c) Dairy products and eggs	-0.325	-1.304	0.608	0.008	3737	1
(d) Soy products	0.457	-0.686	1.618	0.01	3646	1
(e) Seafood	0.105	-1.076	1.475	0.011	3797	1
(f) Fruit	0.064	-0.94	1.203	0.009	3979	1
(g) Dark-green and yellow vegetables	-0.955	-2.358	0.222	0.012	3282	1
(h) Other vegetables, mushrooms, etc.	-0.487	-1.721	0.787	0.012	3346	1
(i) Bread, noodles, and potatoes	0.82	-0.516	2.088	0.011	3806	1
(j) Confectionery and seasonings, etc.	-0.167	-1.42	0.991	0.01	4275	1
Females × (a) Meat	0.111	-1.3	1.489	0.011	4532	1
Females × (b) Processed meat and fish products	0.424	-0.822	1.768	0.01	4736	1
Females × (c) Dairy products and eggs	0.233	-0.785	1.366	0.01	3501	1
Females × (d) Soy products	0.532	-0.764	1.782	0.011	4067	1
Females × (e) Seafood	-0.245	-1.549	1.168	0.011	4605	1
Females × (f) Fruit	-0.409	-1.653	0.874	0.01	4407	1
Females × (g) Dark-green and yellow vegetables	0.324	-0.974	1.709	0.011	4142	1
Females × (h) Other vegetables, mushrooms, etc.	0.365	-1.045	1.67	0.011	4362	1
Females × (i) Bread, noodles, and potatoes	0.321	-1.024	1.701	0.011	4363	1
Females × (j) Confectionery and seasonings, etc.	-0.44	-1.81	0.857	0.01	5096	1
Age	0.743	-0.113	1.59	0.007	4391	1
BMI	-0.267	-1.011	0.463	0.006	4548	1
University	-0.112	-1.242	1.163	0.008	5777	1
Cohabitants	-0.949	-2.199	0.26	0.008	6158	1
Employment	-0.609	-1.95	0.578	0.009	6080	1
Activity L	-0.166	-1.462	1.159	0.009	6119	1
Activity H	-0.718	-2.055	0.635	0.01	5094	1

Regressand: Irritability

Regressor	Mean	HDI 3%	HDI 97%	MCSE Meran	ESS Bulk	R-hat
(a) Meat	0.378	-0.906	1.546	0.01	3978	1
(b) Processed meat and fish products	-0.203	-1.334	0.872	0.01	3763	1
(c) Dairy products and eggs	0.468	-0.494	1.404	0.008	4079	1
(d) Soy products	0.122	-1.026	1.256	0.01	3720	1
(e) Seafood	0.196	-1.055	1.442	0.01	4144	1
(f) Fruit	-0.083	-1.079	0.916	0.008	4787	1
(g) Dark-green and yellow vegetables	0.019	-1.158	1.303	0.011	3602	1
(h) Other vegetables, mushrooms, etc.	0.202	-1.053	1.395	0.011	3594	1
(i) Bread, noodles, and potatoes	-0.025	-1.426	1.228	0.012	3709	1
(j) Confectionery and seasonings, etc.	0.098	-1.132	1.306	0.01	4441	1
Females × (a) Meat	-0.796	-2.252	0.567	0.012	4043	1
Females × (b) Processed meat and fish products	0.23	-1.074	1.419	0.01	4566	1
Females × (c) Dairy products and eggs	-0.436	-1.48	0.59	0.009	4212	1
Females × (d) Soy products	-0.408	-1.664	0.871	0.011	4125	1
Females × (e) Seafood	-0.179	-1.539	1.233	0.012	4020	1
Females × (f) Fruit	-0.321	-1.593	0.773	0.009	5233	1
Females × (g) Dark-green and yellow vegetables	-0.124	-1.424	1.164	0.01	4709	1
Females × (h) Other vegetables, mushrooms, etc.	-0.27	-1.534	1.056	0.011	4379	1
Females × (i) Bread, noodles, and potatoes	0.624	-0.882	1.971	0.012	4315	1
Females × (j) Confectionery and seasonings, etc.	-0.333	-1.554	1.029	0.01	4843	1
Age	0.954	0.153	1.81	0.006	4764	1
BMI	0.418	-0.291	1.089	0.005	5956	1
University	-1.961	-3.111	-0.804	0.008	6000	1
Cohabitants	-1.337	-2.526	-0.12	0.008	6691	1
Employment	-0.622	-2.057	0.598	0.009	6286	1
Activity L	0.328	-0.959	1.577	0.008	6777	1
Activity H	-0.329	-1.721	1.026	0.009	6396	1

Note. Mean: posterior mean. HDI 3% (97%): 3% (97%) quantile of the highest density interval. MCSE Mean: Monte Carlo standard error of the mean. ESS Bulk: effective Sample Size. R-hat: Gelman–Rubin statistic.

Table S4 (Continued). Estimation and convergence diagnostics

Regressand: Fatigue

Regressor	Mean	HDI 3%	HDI 97%	MCSE Meran	ESS Bulk	R-hat
(a) Meat	0.286	-1.037	1.625	0.012	3292	1
(b) Processed meat and fish products	-0.319	-1.491	0.871	0.012	2923	1
(c) Dairy products and eggs	-0.082	-1.05	0.933	0.008	4091	1
(d) Soy products	-0.225	-1.516	0.955	0.013	2718	1
(e) Seafood	0.432	-0.845	1.781	0.013	2840	1
(f) Fruit	0.483	-0.634	1.571	0.01	3529	1
(g) Dark-green and yellow vegetables	-0.257	-1.473	1.049	0.012	3346	1
(h) Other vegetables, mushrooms, etc.	-0.264	-1.395	1.01	0.011	3450	1
(i) Bread, noodles, and potatoes	0.685	-0.645	2.115	0.013	3296	1
(j) Confectionery and seasonings, etc.	0.044	-1.17	1.262	0.01	4339	1
Females × (a) Meat	-0.802	-2.249	0.59	0.012	4127	1
Females × (b) Processed meat and fish products	0.216	-1.076	1.553	0.011	3694	1
Females × (c) Dairy products and eggs	0.312	-0.765	1.396	0.009	3870	1
Females × (d) Soy products	-0.19	-1.54	1.159	0.012	3790	1
Females × (e) Seafood	0.32	-1.174	1.745	0.014	3033	1
Females × (f) Fruit	-0.47	-1.794	0.779	0.011	4006	1
Females × (g) Dark-green and yellow vegetables	-0.744	-2.098	0.589	0.011	4182	1
Females × (h) Other vegetables, mushrooms, etc.	-0.385	-1.665	1.008	0.011	4330	1
Females × (i) Bread, noodles, and potatoes	0.921	-0.504	2.453	0.011	5001	1
Females × (j) Confectionery and seasonings, etc.	0.054	-1.303	1.326	0.01	4474	1
Age	-0.133	-1.012	0.718	0.007	4703	1
BMI	0.431	-0.362	1.207	0.006	4353	1
University	-0.68	-1.911	0.554	0.008	6288	1
Cohabitants	-0.549	-1.732	0.765	0.009	5972	1
Employment	0.022	-1.265	1.36	0.009	6214	1
Activity L	0.167	-1.131	1.525	0.009	6230	1
Activity H	0.124	-1.332	1.383	0.009	6828	1

Regressand: Anxiety

Regressor	Mean	HDI 3%	HDI 97%	MCSE Meran	ESS Bulk	R-hat
(a) Meat	-0.124	-1.401	1.093	0.012	3135	1
(b) Processed meat and fish products	-0.162	-1.277	1.024	0.011	3461	1
(c) Dairy products and eggs	-0.542	-1.602	0.459	0.009	3923	1
(d) Soy products	-0.172	-1.476	1.049	0.011	3840	1
(e) Seafood	0.51	-0.81	1.877	0.011	3993	1
(f) Fruit	0.449	-0.704	1.506	0.009	4674	1
(g) Dark-green and yellow vegetables	-0.335	-1.614	0.963	0.011	3971	1
(h) Other vegetables, mushrooms, etc.	-0.146	-1.36	1.093	0.011	3887	1
(i) Bread, noodles, and potatoes	0.459	-0.846	1.881	0.011	4349	1
(j) Confectionery and seasonings, etc.	-0.06	-1.333	1.111	0.01	4245	1
Females × (a) Meat	-0.324	-1.697	1.118	0.011	4457	1
Females × (b) Processed meat and fish products	0.196	-1.107	1.404	0.011	4013	1
Females × (c) Dairy products and eggs	-0.225	-1.407	0.845	0.009	4410	1
Females × (d) Soy products	0.235	-1.154	1.637	0.012	3999	1
Females × (e) Seafood	-0.144	-1.551	1.372	0.012	4234	1
Females × (f) Fruit	0.141	-1.212	1.456	0.01	5001	1
Females × (g) Dark-green and yellow vegetables	0.036	-1.379	1.466	0.012	4169	1
Females × (h) Other vegetables, mushrooms, etc.	-0.427	-1.782	0.931	0.011	4310	1
Females × (i) Bread, noodles, and potatoes	1.22	-0.252	2.655	0.012	4124	1
Females × (j) Confectionery and seasonings, etc.	0.575	-0.823	1.889	0.011	4706	1
Age	-0.412	-1.198	0.446	0.007	3843	1
BMI	-0.048	-0.824	0.707	0.006	5265	1
University	-0.122	-1.402	1.073	0.008	6330	1
Cohabitants	-0.883	-2.129	0.43	0.009	6028	1
Employment	-0.57	-1.859	0.82	0.01	5220	1
Activity L	-0.147	-1.494	1.273	0.009	7070	1
Activity H	-0.162	-1.498	1.236	0.008	7729	1

Note. Mean: posterior mean. HDI 3% (97%): 3% (97%) quantile of the highest density interval. MCSE Mean: Monte Carlo standard error of the mean. ESS Bulk: effective Sample Size. R-hat: Gelman–Rubin statistic.

Table S4 (Continued). Estimation and convergence diagnostics

Regressand: Depression

Regressor	Mean	HDI 3%	HDI 97%	MCSE Meran	ESS Bulk	R-hat
(a) Meat	0.377	-0.907	1.527	0.01	4444	1
(b) Processed meat and fish products	0.077	-1.087	1.289	0.01	3922	1
(c) Dairy products and eggs	-0.155	-1.217	0.852	0.008	4337	1
(d) Soy products	-0.249	-1.465	0.916	0.011	3485	1
(e) Seafood	0.384	-0.931	1.705	0.01	4629	1
(f) Fruit	0.047	-1.079	1.172	0.009	4236	1
(g) Dark-green and yellow vegetables	-1.106	-2.38	0.157	0.011	4005	1
(h) Other vegetables, mushrooms, etc.	0.242	-1.027	1.485	0.011	3996	1
(i) Bread, noodles, and potatoes	0.845	-0.538	2.224	0.011	4449	1
(j) Confectionery and seasonings, etc.	0.059	-1.176	1.286	0.009	4960	1
Females × (a) Meat	-0.489	-1.865	0.933	0.011	4512	1
Females × (b) Processed meat and fish products	0.828	-0.59	2.119	0.01	5028	1
Females × (c) Dairy products and eggs	0.347	-0.788	1.463	0.009	3998	1
Females × (d) Soy products	0.059	-1.314	1.352	0.01	4848	1
Females × (e) Seafood	0.226	-1.193	1.562	0.012	4248	1
Females × (f) Fruit	-0.512	-1.777	0.746	0.01	4756	1
Females × (g) Dark-green and yellow vegetables	-0.513	-1.866	0.771	0.011	4502	1
Females × (h) Other vegetables, mushrooms, etc.	0.246	-1.092	1.583	0.01	4606	1
Females × (i) Bread, noodles, and potatoes	1.336	-0.128	2.753	0.01	5494	1
Females × (j) Confectionery and seasonings, etc.	0.781	-0.532	2.1	0.01	5287	1
Age	-1.012	-1.874	-0.232	0.006	5062	1
BMI	-0.705	-1.466	0.178	0.006	5368	1
University	-0.573	-1.816	0.702	0.007	8026	1
Cohabitants	-1.574	-2.786	-0.248	0.008	6571	1
Employment	-0.378	-1.684	0.894	0.008	6839	1
Activity L	-0.142	-1.582	1.172	0.008	7598	1
Activity H	-0.835	-2.293	0.559	0.009	7424	1

Regressand: Physical

Regressor	Mean	HDI 3%	HDI 97%	MCSE Meran	ESS Bulk	R-hat
(a) Meat	-0.564	-1.756	0.787	0.011	3811	1
(b) Processed meat and fish products	-1.409	-2.59	-0.313	0.01	3717	1
(c) Dairy products and eggs	0.317	-0.662	1.229	0.008	4201	1
(d) Soy products	0.332	-0.824	1.643	0.012	3200	1
(e) Seafood	0.253	-1.131	1.451	0.01	4399	1
(f) Fruit	-0.249	-1.257	0.858	0.009	3968	1
(g) Dark-green and yellow vegetables	0.365	-0.867	1.567	0.011	3716	1
(h) Other vegetables, mushrooms, etc.	0.234	-0.924	1.474	0.01	4319	1
(i) Bread, noodles, and potatoes	0.8	-0.531	2.175	0.012	3896	1
(j) Confectionery and seasonings, etc.	0.157	-1.039	1.383	0.01	4524	1
Females × (a) Meat	0.251	-1.121	1.765	0.011	4561	1
Females × (b) Processed meat and fish products	-0.024	-1.209	1.319	0.01	4218	1
Females × (c) Dairy products and eggs	-1.001	-2.127	0.098	0.009	4376	1
Females × (d) Soy products	-0.351	-1.733	1.061	0.011	4658	1
Females × (e) Seafood	-0.083	-1.569	1.295	0.012	4037	1
Females × (f) Fruit	0.044	-1.148	1.408	0.01	4833	1
Females × (g) Dark-green and yellow vegetables	-0.697	-2.049	0.625	0.011	3928	1
Females × (h) Other vegetables, mushrooms, etc.	0.059	-1.253	1.434	0.011	4374	1
Females × (i) Bread, noodles, and potatoes	0.8	-0.614	2.278	0.012	4164	1
Females × (j) Confectionery and seasonings, etc.	0.102	-1.114	1.535	0.01	4941	1
Age	0.043	-0.791	0.857	0.006	4632	1
BMI	0.18	-0.593	1.013	0.005	6104	1
University	-0.393	-1.668	0.807	0.008	5911	1
Cohabitants	-0.359	-1.684	0.871	0.009	6286	1
Employment	-1.441	-2.758	-0.058	0.009	5961	1
Activity L	0.064	-1.221	1.409	0.009	6883	1
Activity H	-0.168	-1.521	1.259	0.009	6737	1

Note. Mean: posterior mean. HDI 3% (97%): 3% (97%) quantile of the highest density interval. MCSE Mean: Monte Carlo standard error of the mean. ESS Bulk: effective Sample Size. R-hat: Gelman–Rubin statistic.

Table S4 (Continued). Estimation and convergence diagnostics

Standard deviation / Correlation coefficient	Mean	HDI 3%	HDI 97%	MCSE Meran	ESS Bulk	R-hat
σ1(Vigor)	2.585	1.448	3.826	0.014	1951	1
σ2(Irritability)	2.252	1.158	3.412	0.012	2321	1
σ3(Fatigue)	3.54	2.499	4.692	0.01	3878	1
σ4(Anxiety)	3.067	1.919	4.151	0.011	2834	1
σ5(Depression)	2.549	1.537	3.643	0.01	3226	1
σ6(Physical)	2.224	1.158	3.317	0.012	2367	1
ρ12(Vigor-Irritability)	0.228	-0.186	0.645	0.006	1314	1
ρ13(Vigor-Fatigue)	0.535	0.255	0.813	0.005	905	1
ρ14(Vigor-Anxiety)	0.379	0.039	0.731	0.006	1040	1
ρ15(Vigor-Depression)	0.589	0.306	0.875	0.004	1693	1
ρ16(Vigor-Physical)	0.265	-0.163	0.687	0.006	1391	1
ρ23(Irritability-Fatigue)	0.616	0.355	0.891	0.004	1598	1
ρ24(Irritability-Anxiety)	0.535	0.218	0.852	0.005	1378	1
ρ25(Irritability-Depression)	0.477	0.107	0.819	0.004	2070	1
ρ26(Irritability-Physical)	0.589	0.269	0.909	0.004	1989	1
ρ34(Fatigue-Anxiety)	0.755	0.559	0.926	0.002	2634	1
ρ35(Fatigue-Depression)	0.747	0.529	0.94	0.002	3161	1
ρ36(Fatigue-Physical)	0.653	0.377	0.896	0.003	2809	1
ρ45(Anxiety-Depression)	0.686	0.439	0.91	0.002	3360	1
ρ46(Anxiety-Physical)	0.605	0.302	0.881	0.003	3180	1
ρ56(Depression-Physical)	0.542	0.202	0.866	0.003	3056	1

Note. Mean: posterior mean. HDI 3% (97%): 3% (97%) quantile of the highest density interval. MCSE Mean: Monte Carlo standard error of the mean. ESS Bulk: effective Sample Size. R-hat: Gelman–Rubin statistic.
